# The conserved sex regulator DMRT1 recruits SOX9 in sexual cell fate reprogramming

**DOI:** 10.1093/nar/gkab448

**Published:** 2021-06-07

**Authors:** Robin E Lindeman, Mark W Murphy, Kellie S Agrimson, Rachel L Gewiss, Vivian J Bardwell, Micah D Gearhart, David Zarkower

**Affiliations:** Developmental Biology Center and Department of Genetics, Cell Biology, and Development, University of Minnesota, Minneapolis, MN 55455 USA; Developmental Biology Center and Department of Genetics, Cell Biology, and Development, University of Minnesota, Minneapolis, MN 55455 USA; Developmental Biology Center and Department of Genetics, Cell Biology, and Development, University of Minnesota, Minneapolis, MN 55455 USA; Developmental Biology Center and Department of Genetics, Cell Biology, and Development, University of Minnesota, Minneapolis, MN 55455 USA; Developmental Biology Center and Department of Genetics, Cell Biology, and Development, University of Minnesota, Minneapolis, MN 55455 USA; University of Minnesota Masonic Cancer Center, Minneapolis, MN 55455, USA; Developmental Biology Center and Department of Genetics, Cell Biology, and Development, University of Minnesota, Minneapolis, MN 55455 USA; Developmental Biology Center and Department of Genetics, Cell Biology, and Development, University of Minnesota, Minneapolis, MN 55455 USA; University of Minnesota Masonic Cancer Center, Minneapolis, MN 55455, USA

## Abstract

Mammalian sexual development commences when fetal bipotential progenitor cells adopt male Sertoli (in XY) or female granulosa (in XX) gonadal cell fates. Differentiation of these cells involves extensive divergence in chromatin state and gene expression, reflecting distinct roles in sexual differentiation and gametogenesis. Surprisingly, differentiated gonadal cell fates require active maintenance through postnatal life to prevent sexual transdifferentiation and female cell fate can be reprogrammed by ectopic expression of the sex regulator DMRT1. Here we examine how DMRT1 reprograms granulosa cells to Sertoli-like cells in vivo and in culture. We define postnatal sex-biased gene expression programs and identify three-dimensional chromatin contacts and differentially accessible chromatin regions (DARs) associated with differentially expressed genes. Using a conditional transgene we find DMRT1 only partially reprograms the ovarian transcriptome in the absence of SOX9 and its paralog SOX8, indicating that these factors functionally cooperate with DMRT1. ATAC-seq and ChIP-seq show that DMRT1 induces formation of many DARs that it binds with SOX9, and DMRT1 is required for binding of SOX9 at most of these. We suggest that DMRT1 can act as a pioneer factor to open chromatin and allow binding of SOX9, which then cooperates with DMRT1 to reprogram sexual cell fate.

## INTRODUCTION

In mammals, sex is typically determined in mid-gestation, shortly after the gonadal primordium, or genital ridge, forms, which occurs around embryonic day 10.5 (E10.5) in mice. Gonadal sex determination is a binary switch that operates in bipotential somatic progenitor cells to trigger their differentiation into either Sertoli cells in males or granulosa cells in females (1). In XY genetic males, expression of the Y-linked gene *Sry* in the bipotential progenitor cells activates the related gene *Sox9* and initiates a cascade of gene regulation leading to testis formation, while in XX genetic females an alternative genetic network involving WNT/β-CATENIN signaling predominates instead and directs ovarian differentiation (2). Once specified, Sertoli and granulosa cells then initiate further downstream regulatory events that induce sexual differentiation of other gonadal cell types and ultimately establish the sex of the entire body as either male or female (3).

RNA analysis has shown that XX and XY bipotential progenitor cells exhibit virtually identical gene expression prior to SRY expression and sex determination but then rapidly diverge, activating a network of genes specifying differentiation of one cell type and repressing genes that would specify the alternative fate (4,5). Coincident with the establishment of these sex-biased gene regulatory networks, somatic precursor cells acquire many sex-biased differences in the chromatin landscape, including ‘open’ regions accessible to regulatory factors (6). As expected, these open regions are enriched for motifs bound by a number of key regulators of sexual differentiation, including SF1, GATA4, DMRT1, SOX8 and SOX9 in XY cells, and SF1, GATA4 and LHX9 in XX cells (6). The onset of sex-biased gene expression that results from the activity of these regulators also is accompanied by a shift in promoter-proximal histone modifications at target genes (6). The combined identification of gene expression changes, chromatin changes, and potential regulatory factor binding sites in the mouse is beginning to allow detailed sex regulatory networks to be constructed for the fetal gonad.

Sexual cell fate in the somatic gonad normally is stable once sex determination has committed bipotential progenitors to the Sertoli or granulosa path during fetal development and differentiation has begun. However, mouse genetics has revealed that this stability relies on continually-active sex maintenance systems that operate in the gonads of both sexes. These maintenance systems can be revealed by deleting a key transcriptional regulator. In the ovary, deletion of *Foxl2* or the estrogen receptor genes *Esr1* and *Esr2* causes granulosa cells to transdifferentiate into Sertoli-like cells (7–9). Conversely, in the testis, loss of *Dmrt1* (10) or loss of *Sox9* together with its close paralog *Sox8* causes Sertoli cells to transdifferentiate into granulosa-like cells (10–13). These cell fate transformations can occur even when *Foxl2* or *Dmrt1* is deleted in the adult gonad, long after ‘terminal’ differentiation and, in the case of *Dmrt1* in Sertoli cells, long after exit from the cell cycle (7,10). It is therefore clear that DMRT1, FOXL2, and likely other transcription factors are essential components of opposed cell fate maintenance networks whose sustained activity is necessary in each sex to keep sexual cell fate ‘locked-in’ throughout postnatal life.

DMRT1 (doublesex and mab-3 related transcription factor 1) is part of a deeply conserved family of sex regulators (14,15). These proteins share a distinct zinc-binding DNA recognition module, the DM domain, and family members regulate sexual development across most metazoans, making them the most ancient and widespread sex regulators known (15). As such, their regulatory mechanisms are of significant interest. DMRT1 has a unique mode of DNA interaction involving insertion of recognition helices from two protomers together into the DNA major groove and can bind DNA in at least three stoichiometries (16). How the unusual DNA binding modality of DMRT1 affects its transcriptional regulatory properties is not yet understood.

DMRT1 not only is necessary in mice for male sex maintenance, but also is sufficient to induce female-to-male *in vivo* cell fate reprogramming. Conditional expression of *Dmrt1* in XX fetal somatic gonad cells, driven by *Sf1-Cre* from around the time of sex determination, can activate expression of *Sox8, Sox9* and other male regulators starting about two weeks postnatally, and leads to formation of seminiferous tubule-like structures lined with polarized SOX9-expressing cells with typical adult Sertoli morphology (17). This morphological remodeling of the gonad is accompanied by a molecular remodeling of the ovarian transcriptome toward a more testis-like state. Thus DMRT1 not only is essential for maintenance of male sexual fate in the postnatal testis, but also is able to act instructively to impose male fate and suppress female fate in the postnatal ovary.

A number of other cell fate reprogramming examples have been described, but most have involved cultured cells, activation of multiple reprogramming factors, or both (18–22). Manipulation of sexual cell fate by altering DMRT1 or FOXL2 expression stands out as a rare example where altering the activity of a single regulator can reprogram fully differentiated cells *in vivo*. Moreover, because its gain or loss is sufficient to toggle cell fate in either direction, DMRT1 presumably occupies a pivotal position in the postnatal sex regulatory network.

Sexual fate reprogramming by DMRT1 may therefore provide a useful paradigm for cell fate control, but a number of important mechanistic questions remain to be addressed. One question is whether the genes and regulatory elements involved in reprogramming extensively overlap with those used to establish sexual cell fate during normal development. Another question is how ectopic DMRT1 interacts with the granulosa cell chromatin landscape to cause male gene expression to replace the female program. A third question regards the relative contributions of DMRT1 and SOX9 to granulosa-to-Sertoli cell reprogramming. Ectopic SOX9 expression during a fetal period when DMRT1 is expressed in both sexes can direct bipotential progenitors in XX gonads to develop as Sertoli cells (23,24). In contrast, ectopic expression of DMRT1 in the fetal gonad does not activate SOX9 or disrupt fetal granulosa cell specification (17). Postnatally, however, ectopic DMRT1 expression activates SOX9 and can reprogram granulosa cells into Sertoli-like cells (17). It therefore is possible that SOX9 plays a major role in postnatal reprogramming of XX granulosa cells to Sertoli-like cells and does so in cooperation with DMRT1. However, it is unknown whether either DMRT1 or SOX9 can reprogram sexual cell fate independently.

Here, we investigate the mechanism of granulosa-to-Sertoli reprogramming and the roles of DMRT1 and SOX9 in that process. We first identify genes with Sertoli- and granulosa-biased postnatal expression and show that many are associated with sex-biased differentially-accessible chromatin regions (DARs). The tendency of DARs preferentially accessible in one sex to be located near genes that are more active in that sex suggests that postnatal sexual cell fates rely more on positive than negative transcriptional regulation. Using a conditional expression system, we find that DMRT1 can shift the postnatal ovarian transcriptome toward a more testis-like state even in the absence of SOX8/9. However, many genes cannot be fully regulated by DMRT1 without either SOX9 or its paralog SOX8, suggesting that DMRT1 and SOX8/9 have cooperative roles. Consistent with this idea, we find that DMRT1 and SOX9 bind jointly at a subset of sex-biased accessible regions. Using cultured primary granulosa cells we show that chromatin accessibility and binding by SOX9 to many of its target sites requires DMRT1, suggesting that DMRT1 may act as a pioneer factor to open chromatin for SOX9 and potentially other regulators. Given the deeply conserved roles of DMRT1 and SOX9 in vertebrate sexual development, we suggest that this collaborative mode of gene regulation may extend beyond mammals.

## MATERIALS AND METHODS

### Mice

*CAG-Stop^flox^-Dmrt1-eGfp* mice are described in (17). *CAG*-*mRFP1^floxed^*-*Sox9*-*EGFP* mice are described in (25) and were a gift from R. Behringer. *CAG-CreER^TM^* and *CAG-Stop^flox^-tdTomato* mice were purchased from Jackson Laboratories (stock numbers 004682 and 007914). Animals homozygous or heterozygous for the *CAG-Stop^flox^-Dmrt1-eGfp* or *CAG*-*mRFP1^floxed^*-*Sox9*-*eGFP* transgenes were bred to mice carrying the *CAG-CreER* to generate experimental animals. Whenever possible, littermate controls lacking the *Cre* and/or the *Sox9/Dmrt1* transgene were used; otherwise age-matched wild-type animals were used as controls. *Sf1-Cre* mice (26) were provided by K. Parker, *Dhh-Cre* mice (27) were provided by D. Meier, *UBC-CreERT2* mice (28) by E. Brown, *Hsd17b1-CreERT2* mice (29) by E. Casanova, *Sox9^flox/+^* mice (30) by R. Behringer, and *Sox8^+/^^−^;Sox9^flox/+^* mice (31) by M. Wegner. *Dmrt1^flox/+^* mice are described in (32). Mice were of mixed genetic background (129Sv and C57Bl/6J) and maintained in conventional housing facilities. Presence of a copulation plug in the morning was recorded as day E0.5. Experimental protocols were approved by the University of Minnesota Animal Care and Use Committee.

### Genotyping

PCR genotyping on tail-clip DNA for the *CAG-Stop^flox^-Dmrt1-eGfp* transgene was conducted as previously described (17). Genotyping for *CAG*-*mRFP1^floxed^*-*Sox9*-*eGFP* used primers mRFP F (5′ TCCCCGACTACTTGAAGCTG 3′) and mRFP R (5′ CTTGGCCATGTAGGTGGTCT 3′), which yield an approximately 320 bp DNA product. Reactions ran 32 cycles with a 30 second (s) 58°C annealing step and 40 s 72°C elongation. *Sox8* genotyping was conducted using primers RL11 (5′ GTCCTGCGTGGCAACCTTGG 3′), RL12 (5′ GCCCACACCATGAAGGCATTC 3′) and RL13 (5′ TAAAAATGCGCTCAGGTCAA3′), which yield a 430 bp DNA product from the WT allele and 617 bp from the null allele. *Sox9* genotyping was conducted using primers RL9 (5′ CCGGCTGCTGGGAAAGTATATG 3′), RL10 (5′ CGCTGGTATTCAGGGAGGTACA 3′), and RL10.5 (5′ CTCCGGTAGCAAAGGCGTTTAG 3′), which yield DNA products of 247 bp from WT and 419 bp from the floxed allele. Reactions for *Sox8* and *Sox9* ran 35 cycles with a 30 s 56°C annealing step and 45 s 72°C elongation. Primers and amplification conditions for genotyping the *Cre* transgenes and *Dmrt1* floxed and deleted alleles are described in (32).

### Granulosa cell culture

Primary granulosa cell isolation was performed following the follicle puncture method (33). In summary, ovaries were collected from 23- to 29-day-old mice and transferred to petri dishes (60 × 15 mm) containing phosphate-buffered saline (PBS). Ovaries of the same genotype were pooled. The surrounding connective tissue, fat, and bursa were removed using forceps and ovaries were washed once in fresh PBS. Ovaries were then transferred to small (35 × 10 mm) petri dishes containing 2.5 ml pre-warmed McCoy 5A medium (Sigma) with 6.8 mM ethylene glycol tetraacetic acid (EGTA) and 26 mM sodium bicarbonate, and were incubated at 37°C for 10 min. Ovaries were then transferred to a new dish containing 2.5 ml pre-warmed 0.5 M sucrose in McCoy 5A medium and incubated at 37°C for 5 min. For cell collection, ovaries were placed into a fresh dish containing 2.5 ml pre-warmed serum-free McCoy's 5A medium. A 27G needle was then used to puncture the ovarian follicles, concentrating on the medium-to-large follicles at the periphery of the ovary. Ovaries were gently squeezed with forceps to expel the granulosa cells. The punctured ovary tissues were then removed and discarded. The cells remaining in the dish were resuspended by pipetting and transferred to a 15 ml conical tube. Cells were pelleted at 500 × g for 7 min and the cell pellet was resuspended in an appropriate volume of complete media (McCoy 5A medium with 10% fetal bovine serum (FBS), 1× Pen/Strep). Cells were then plated in Falcon six well (Life Sciences 353046) 35 × 10 mm tissue culture dishes (two ovaries/well) and incubated overnight at 37°C in a humidified incubator with 5% CO_2_.

For experiments involving induction of DMRT1 or SOX9 in cultured granulosa cells, tamoxifen treatments began on day 2 of culture (24 h after plating). Immediately prior to treatment, 2 mM 4-hydroxytamoxifen (in ethanol; Sigma H7904) was diluted to 2 μM in serum-free McCoy 5A with 0.1% BSA, 1× ITSS supplement (Sigma I1884) and 1× Pen/Strep. Cells were tamoxifen-treated for 48 hours before replacing media with complete media. Thereafter, media was changed every 2–3 days. Total culture time, except for time courses, was 7 days (1 day without tamoxifen plus six days with tamoxifen).

### Sertoli cell isolation

Testes were dissected from P7 mice carrying *CAG-Stop^flox^-tdTomato* driven by *Dhh-Cre*. Testis tubules were manually dissected and loosened with forceps and suspended in 800 ul PBS with four tubule bundles (from two mice) per 2 ml microfuge tube. Then, 50 μl Col1A (5 mg/ml; Sigma C5894) followed by 200 μl fetal bovine serum and 1 μl DNAse (10 mg/ml, Roche 10104159001) were added and tubules were incubated at 37°C for 10 min with rotation. After incubation, tubules were allowed to settle, then were rinsed 3–6 times with PBS, centrifuging the tubules for 5 s at 50 × g between each subsequent rinse. The rinsed tubule pellet was resuspended in 200 ul trypsin/EDTA with pipetting to break up any remaining clumps and incubated at 37°C for 10 min with rotation, and then an additional 600 ul trypsin/EDTA was added followed by an additional 10 min incubation at 37°C with rotation. 50 ul DNAse (10 mg/ml) was added to each tube followed by a 5 min incubation at 37°C with rotation. Cells were then filtered through a 40 um mesh, pelleted at 1500 rpm in a clinical centrifuge, transferred to a 2 ml microfuge tube, and resuspended in 300 ul PBS with 0.5% FBS. Next, 5 μl THY1 and 5 μl c-KIT antibody beads (Miltenyi Biotec, 130-049-101 and 130-091-224) were added followed by a 15 min incubation at 4°C and the cell mixture was applied to an equilibrated LS separation column (Miltenyi, 130-042-401) on a Miltenyi magnetic stand. Flow-through, enriched for Sertoli cells, was collected along with a 1 ml rinse. Cells were examined by epifluorescence microscopy to determine the number and purity of tdTomato-positive Sertoli cells, and all cell preparations used were at least 90% pure.

### Immunofluorescent staining

Primary granulosa cells were grown on ethanol-sterilized round glass coverslips in four-well plates (Thermo Scientific 176740) in 0.5 ml media. At the designated time points, coverslips were removed to fresh dishes and washed once in PBS, then fixed for 20 min at room temperature (RT) in 4% paraformaldehyde. Coverslips were then washed three times in PBS and stored in PBS with 0.1% bovine serum albumen (BSA) until staining. Antigen retrieval was performed using 95°C citric acid buffer for 10 min followed by a 10 min permeabilization in PBS with 0.1% Triton X-100. Cells were blocked in PBS with 3% BSA, 5% donkey serum and 0.1% Triton-X for 1 h at RT. Coverslips were then incubated in the diluted primary antibodies (rabbit anti-DMRT1 (34) at 1:500, goat anti-GFP at 1:500 (Novus NB100-1770), rabbit anti-SOX9-CT (gift of F. Poulat) at 1:500, goat anti-FOXL2 (AVIVA ARP39574) at 1:200) overnight at 4°C as previously described (17). Coverslips were washed three times in PBS then incubated in diluted secondary antibodies for 1 h at RT in the dark. Secondary antibodies were from Invitrogen: donkey anti-rabbit Alexa Fluor 488 (A21206); Alexa Fluor 594 (A21207); donkey anti-goat Alexa Fluor 488 (A11055) and Alexa Fluor 596 (A11058), all at 1:500. Cells were washed three times, nuclear counter-stained with 4′,6-diamidino-2-phenylindole (DAPI), then mounted on microscope slides. Images were captured with a Zeiss Imager Z1 microscope and Zeiss MRm camera. A Zeiss Apotome structured illumination system was used for higher magnification images. Images were processed using Zeiss Axiovision software and further processed in Adobe Photoshop. ImageJ was used for conducting counts of stained nuclei as described for automatic counting (https://digital.bsd.uchicago.edu/docs/cell_counting_automated_and_manual.pdf). Antibodies used are described in (10,13). The rabbit SOX9-NT antibody used in the *Sox9* KO experiments (35) was a gift from F. Poulat. Immunofluoresence experiments were performed using a minimum of three independent replicates.

### mRNA sequencing

Primary granulosa cells were grown in 12-well tissue culture plates (Corning 3514). At designated time points, cells from two wells were harvested into 1 ml Trizol reagent and stored at –80°C. Total RNA was prepared according to Trizol reagent manufacturer's protocols and further cleaned up using RNAeasy kit protocols and reagents (Qiagen). RNA was quantified via Qubit RNA assay, and 500 ng total RNA per sample were used in stranded mRNA-seq library preparation (KAPA Biosystems, KK8481) for Illumina sequencing. Two to six replicates were performed for RNA-Seq experiments, as tabulated in Supplemental Table S1. RNA-Seq gene counts and FPKM expression values for all RNA-seq samples are summarized in Supplemental Table S3. Gene expression changes and statistics for RNA-Seq experiments are listed in Supplemental Table S4.

### ChIP-seq and ATAC-seq

For ChIP-seq, freshly isolated Sertoli cells or 7 days cultured 4-hydroxytamoxifen-treated granulosa cells were fixed with 1% methanol-free formaldehyde in PBS (Thermo scientific #28906) for 5 min at RT with rotation. Fixation was stopped by addition of glycine to 0.125 M and rotation at RT for 10 min. After pelleting, cells were washed in PBS and stored at –80°C until use. Sertoli cell ChIP was performed with 1–2.5 million cells and granulosa cell ChIP with about 1 million cells. Nuclei were prepared from the fixed cells and chromatin was sheared to an average size of 300–400 bp in a Covaris S220 according to the manufacturer's recommendations. After shearing, the lysate was diluted 1:3 with complete DOC RIPA (36) and the sample was centrifuged at 21 000 × g for ten min at 4°C to pellet any insoluble material. The supernatant was transferred to a fresh siliconized tube and the ChIP was initiated with the addition of ∼1 ug of the relevant antibody and rotated at 4°C overnight. Samples were then spun at 21 000 × g for 10 min to pellet insoluble material and supernatant was mixed with 20 ul Protein A Dynabeads (Invitrogen, 10002D) previously blocked with BSA and yeast tRNA to inhibit non-specific binding. After incubation at 4°C for 60 min with rotation to allow association of protein A with the antibody, beads were applied to a magnet and washed sequentially as described previously (36). After elution and cross link reversal, sequencing libraries were prepared using the Hyper Prep Kit (KAPA Biosystems, KK8502).

Whole gonad ChIP-seq was performed essentially the same as for primary cells ChIP-seq except gonads from two to three animals were pooled and fixed in 1% paraformaldehyde (Electron Microscopy Sciences #15710) for 10 min (ESR2, FOXL2 and DMRT1) or fixed in 2 mM disuccinimidyl glutarate (DSG, ThermoFisher Scientific #20593) at RT for 45 min followed by 1% paraformaldehyde fixation at RT for 15 min (SOX9). After quenching the fix with 0.125M glycine, chromatin was sheared to an average size of 300–500 bp using a tip sonicator. Antibodies were the same ones used for immunofluorescence plus anti-ESR2 (AVIVA ARP37039) and anti-H3K27ac (Abcam ab4729). Two independent replicates were performed for ChIP-seq experiments, except as indicated in Supplemental Table S1. Differential ChIP peaks and statistics are listed in Supplemental Table S2.

ATAC-seq was performed using 50 000 freshly isolated Sertoli or granulosa cells or 7-day cultured tamoxifen-treated cultured granulosa cells. Transposition and library prep were performed as described (37). Two independent replicates were performed for each experiment using freshly isolated cells and three replicates were performed for experiments using cultured cells. ATAC-seq peaks and statistics are listed in Supplemental Table S2.

### Hi-C

Two independent replicates of Hi-C were performed for each cell type on about 1 million primary Sertoli or granulosa cells using the Arima Hi-C Kit (#A510008). Cells were fixed in 2% methanol-free formaldehyde in PBS at RT for 10 min with occasional mixing then quenched by addition of glycine to 0.125 M with occasional mixing for 5 min. Cells were placed on ice for 15 min prior to washing with 1× PBS and stored at –80°C. Hi-C was performed according to Arima Genomics (Document part # A160134 v00). Sequencing libraries were prepared using the KAPA Hyper Prep Kit and xGen Duplex Seq adapters (Integrated DNA Techologies) according to Arima Genomics (Document part # A160139 v00). Sertoli-called and granulosa-called 3D contacts are listed in Supplemental Table S3.

### Bioinformatic analysis

High-throughput Illumina sequencing was performed by the University of Minnesota Genomics Center or GENEWIZ (South Plainfield, NJ) using a combination of HiSeq 2500, HiSeq 4000 and NovaSeq platforms. For each of the experiments, reads were trimmed using Trim Galore (v0.6.0) and cutadapt (v1.18) and assessed for quality with FastQC (v0.11.8). STAR (v2.7.2a), BWA mem (v0.7.12) and HiC-Pro (v2.11.4) were used to map transcriptomic (RNA-seq), genomic (ChIP-seq, ATAC-seq) and 3D (Hi-C) trimmed reads to the GRCm38 (mm10) genome, respectively. The GENCODE M23 gene annotation set was used to estimate strand-specific gene expression data in the RNA-seq data with the Bioconductor package RSubread (v1.28.1) and to assign the locations of nearby genes in the ATAC-seq datasets. For ChIP-seq and ATAC-seq experiments, duplicated reads were removed with Picard MarkDuplicates (v2.17.10) and low quality reads (MAPQ < 55) or reads that mapped to the mitochondrial genome were removed with SAMtools (v1.0). MACS2 (v2.1.1.20160309) was used to call peaks in the ChIP-seq and ATAC-seq datasets using the parameters ‘-call-summits -m 2 20’ and ‘–nomodel –broad’ respectively. Enriched DNA binding motifs were identified using MEME (v5.0.1) using the parameters ‘-maxsize 900000000 -searchsize 0 -text -dna -revcomp -nmotifs 3 -p 1 -nostatus’. Raw fastq files from Garcia-Moreno *et al.* (6) (GSE118755) were downloaded and reprocessed in order to compare postnatal ATAC-seq with embryonic ATAC-seq datasets. For direct comparisons all ATAC-seq datasets were trimmed to 50 bp single-end reads prior to mapping but all of the available data was used for peak calling and figures. Differentially Accessible Regions (DARs) and differentially expressed genes were identified using DESeq2 (v1.26.0). Hi-C data was processed using the LIGATION_SITE = GATCGATC,GATCGANTC,GANTCGATC,GANTCGANTC and GET_PROCESS_SAM = 1 options within HiC-Pro. Contact matrices were normalized and converted to cool format with cooler (v0.8.10) and visualized with HiGlass (v1.9.4). Statistically enriched contact interactions and A/B compartments were identified with cooltools (v0.3.2). Figures were made using custom R scripts which can be downloaded from https://github.com/micahgearhart/granulosa, which contains all informatic workflows. Analyzed genomic data (RNA-seq, ChIP-seq, Hi-C, ATAC-seq) are available in Supplemental Tables S1–S4, with Supplemental Table S1 containing an overall Table of Contents.

## RESULTS

### Potential regulatory elements for postnatal Sertoli versus granulosa cell fate

**Figure 1.**
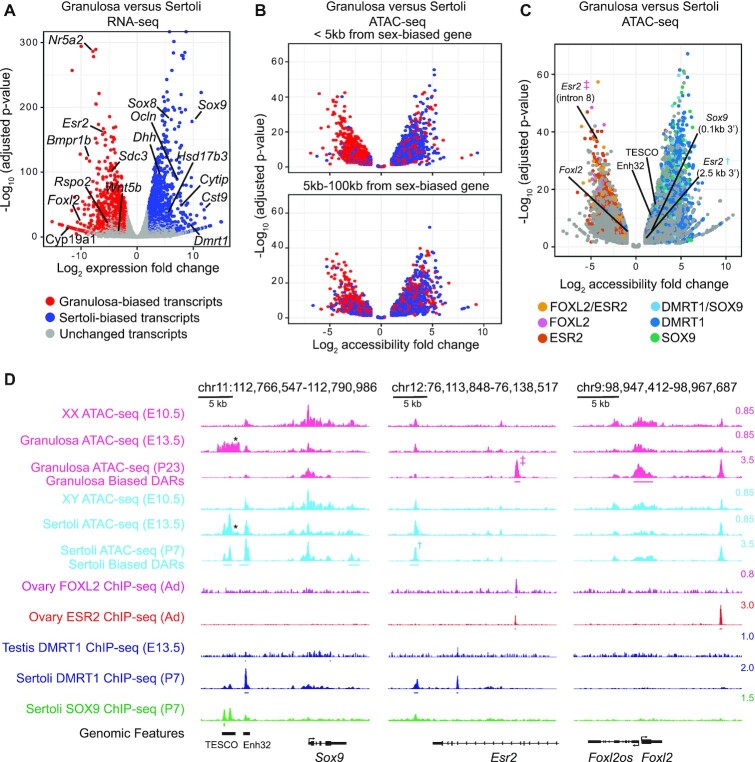
Postnatal sex-biased gonadal gene expression, chromatin accessibility, transcription factor binding and chromatin architecture. (**A**) Plot of RNA-seq data comparing expression differences between postnatal granulosa and Sertoli cells isolated at P23-29 and P7, respectively. mRNAs with significant expression differences are colored red (granulosa-biased) or blue (Sertoli-biased). X axis indicates the log_2_ fold change in expression between the cell types and Y axis corresponds to the –log_10_ of the Benjamini–Hochberg corrected *P*-value such that genes with greater statistical significance are higher on this plot. (**B**, **C**) Plots of ATAC-seq data comparing chromatin accessibility between postnatal granulosa and Sertoli cells. Genomic regions that had a two-fold change in accessibility, a Benjamini-Hochberg adjusted *P*-value <0.05 and a peak-width normalized FPKM value >2.5, or constitutive regions called in both cell types with a fold-change less than 1.25 and an FPKM >2.5 in both cell types are shown. Axes represent the fold-change and significance as in (A). Granulosa-biased differentially accessible regions (DARs) have negative log_2_ values and Sertoli-biased DARs have positive values (X axis). In (B), genomic regions within 5 kilobases (kb) or between 5 and 100 kb of a sex-biased gene are shown in the upper or lower panels respectively. In (C), colors identify DARs bound by DMRT1 and/or SOX9 in Sertoli cells and FOXL2 and ESR2 in the ovary, based on ChIP-seq analysis. DARs labeled with ‡ and † correspond to the peaks labeled in panel D. (**D**) Genomic analysis of regions near *Sox9* (left), *Esr2* (center), and *Foxl2* (right), showing sex-biased ATAC-seq accessibility and transcription factor ChIP-seq. The scale shown at right of each track indicates the number of reads per million reads sequenced for the full height of the track. The locations of DARs and ChIP peaks are indicated by a line underneath each peak for visibility. Prenatal ChIP-seq and ATAC-seq data are from Krentz *et al.* (42) and Garcia-Moreno *et al.* (6) respectively. Note that fetal ATAC-seq signal around TESCO in both sexes (labeled with *) is thought to derive in part from reporter transgene used in cell sorting (38). Coordinates in the GRCm38 genome build are shown at the top of each panel and gene models are diagrammed at bottom.

To learn more about how cell fate is controlled in postnatal Sertoli and granulosa cells we first compared gene expression and chromatin accessibility in the two cell types. RNA-seq analysis identified 872 mRNAs expressed more highly in isolated Sertoli cells and 890 expressed more highly in isolated granulosa cells (>4-fold enrichment, Benjamini-Hochberg adjusted *P*-value <0.05, and mean FPKM >2.5 in the respective cell type; cell isolation described in Materials and Methods). These data defined two groups of postnatal sex-biased genes, which included many genes previously found to be enriched in either Sertoli cells or granulosa cells at other developmental stages (Figure 1A). To identify potential sex-biased regulatory elements controlling these genes we used ATAC-seq to locate regions that differ in chromatin accessibility between postnatal Sertoli and granulosa cells. ATAC-seq identified 19 775 Sertoli-biased and 11 749 granulosa-biased differentially accessible regions (DARs) that had a >2-fold change in accessibility between cell types, a Benjamini–Hochberg adjusted *P*-value <0.05 and mean log_2_ FPKM >2.5 in the respective cell type when using peak width to normalize for the size of each genomic region. Differentially expressed genes and DARs are listed in Supplemental Tables S4 and S2.

To help assess the potential of the sex-biased DARs to mediate sex-biased gene expression, we examined association of DARs with genes showing sex-biased expression (Figure 1B). In particular, we asked whether sex-biased DARs are preferentially associated with genes showing expression biased to that sex, suggesting a positive role in gene regulation. Indeed, the sex-biased genes within 5 kb of Sertoli-biased DARs were predominantly male-biased in their expression (blue dots), while those within 5 kb of granulosa-biased DARs were predominantly female-biased (red dots), as predicted if the DARs play a positive regulatory role in sex-biased gene expression. Specifically, of the 19 775 total Sertoli-biased DARs, 1374 (7%) are within 5 kb of a Sertoli-biased transcript unit and 494 (2%) are within 5 kb of a granulosa-biased transcription unit (Figure 1B, upper panel). Similarly, of the 11 749 granulosa-biased DARs, 1066 (9%) are within 5 kb of a granulosa-biased transcription unit, while 384 (3%) are within 5 kb of a Sertoli-biased transcription unit (Figure 1B, upper panel). A similar, but weaker association was observed at more distal DARs located between 5 kb and 100 kb from sex-biased genes (1492 (8%) Sertoli-biased versus 1032 (5%) granulosa-biased for the Sertoli-biased DARs and 1080 (9%) granulosa-biased versus 691 (6%) Sertoli-biased for the granulosa-biased DARs; Figure 1B, lower panel). These associations suggest that positive and negative regulation both play substantial roles in sex-biased gene expression. For example, there were 486 Sertoli-biased and 235 granulosa-biased genes within 5 kb of Sertoli-biased DARs, suggesting that activation of Sertoli-biased genes. Similarly, among the sex-biased genes near granulosa-biased DARs, 409 were granulosa-biased and 225 were Sertoli-biased, again suggesting both positive and negative regulation.

### Regulatory factor binding to sex-biased DARs

The sex-biased DARs are likely candidates to be bound by sexual fate regulators. To assess this possibility we examined binding of the male sex regulators DMRT1 and SOX9 by ChIP-seq in isolated Sertoli cells and the binding of female sex regulators FOXL2 and ESR2 by ChIP-seq in intact ovaries. A substantial proportion of Sertoli-biased DARs (4397 of 19 775; 22%) were bound by DMRT1, SOX9 or both. Similarly, 18% of granulosa-biased DARs were bound by FOXL2, ESR2 or both (2091 of 11 749) (Figure 1C). The proportion of Sertoli-biased DARs occupied by DMRT1 and SOX9 is consistent with DMRT1 and SOX9 acting through these regions to maintain Sertoli cell fate, either by activating Sertoli-biased genes or repressing granulosa-biased genes.

Three examples of regions with sex-biased expression are shown in Figure 1D. The first region (Figure 1D, left) contains the male-biased *Sox9* gene. ATAC-seq identified three male-biased DARs, one downstream and two upstream of the *Sox9* coding region. The two upstream DARs correspond to two previously identified male-biased enhancer elements, TESCO and Enh32 (38,39). TESCO is bound by both DMRT1 and SOX9 and Enh32 is bound by DMRT1, potentially allowing these two regulators to jointly regulate *Sox9* transcription. The second region (Figure 1D, middle) contains the female-biased *Foxl2* gene and antisense non-coding RNA gene *Foxl2os*. Genome-wide ATAC-seq and ChIP-seq identified a female-biased DAR overlapping the transcriptional start sites, as well as a sex non-specific accessible region downstream of *Foxl2* that is bound by the estrogen receptor ESR2 in the ovary. While the ESR2-bound region is accessible in both sexes, the female-biased expression of ESR2 and the functional requirement of ESR2 for estrogen likely render the activity of this element female-biased. A third region (Figure 1D, right) contains *Esr2*. *Esr2* has a granulosa-biased DAR that forms after E13.5, is bound by FOXL2 and ESR2 in the ovary, and was previously shown to mediate FOXL2-dependent activation of *Esr2* in cultured granulosa-derived cells (40). A region 2.5 kb downstream of *Esr2* contains a Sertoli-biased DAR bound by DMRT1, suggesting activation of *Esr2* expression by FOXL2 and ESR2 in the ovary and repression by DMRT1 in the testis. These examples further illustrate how sex-biased DARs and transcription factor occupancy correlate with sex-biased gene expression. Four additional examples of regions containing sex-biased genes (female-biased *Sdc3* and *Cyp19a1*, and male-biased *Hsd17b3* and *Ocln*) are shown in Supplemental Figure S1.

### Association of putative sex regulatory elements with sex-biased gene expression

**Figure 2.**
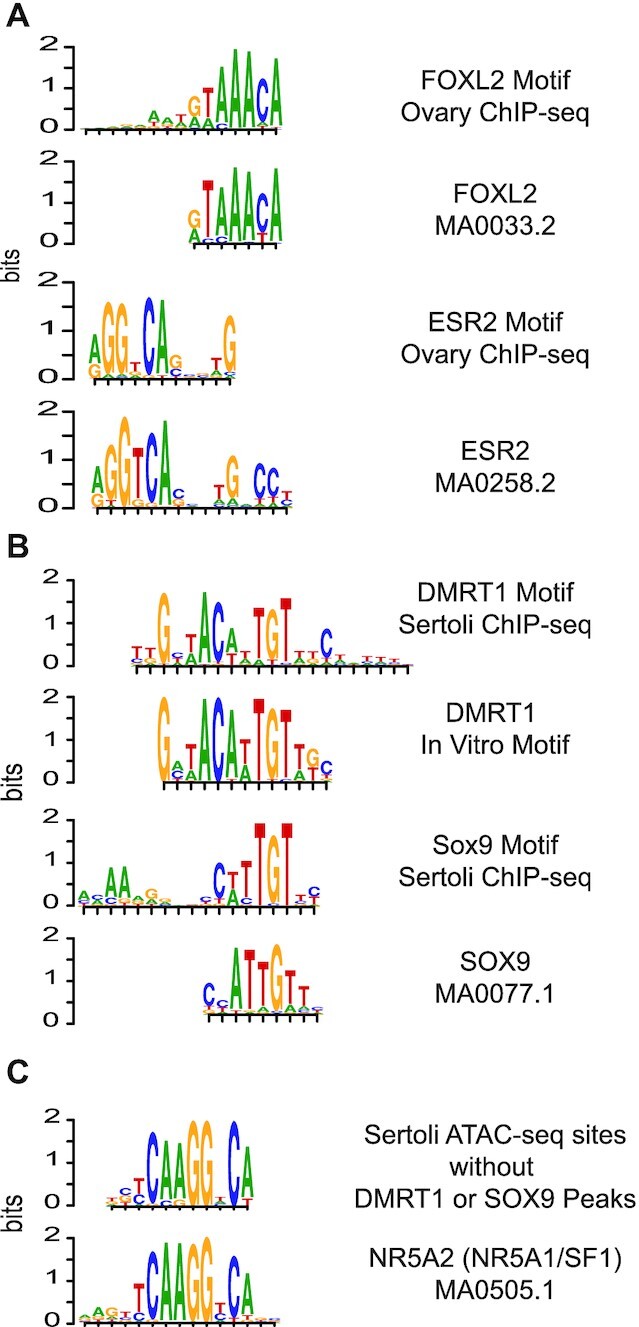
Motifs enriched in ChIP-seq and ATAC-seq analysis. (**A**) Motif enriched in FOXL2 and ESR2 ChIP-seq in adult ovary defined by MEME (upper in each) and reference motif from JASPAR (bottom in each). Each motif was ranked as the top motif in their respective MEME searches and the E-value scores were 1.8e–341 and 3.3e–466 for FOXL2 and ESR2 respectively. (**B**) Motifs enriched in DMRT1 and SOX9 P7 Sertoli cell ChIP-seq above reference motifs from Murphy *et al.* (64) and JASPAR. Each motif was ranked as the top motif in their respective MEME searches and the *E*-value scores were 2.6e-806 and 7.4e–711 for DMRT1 and SOX9 respectively. All four motifs were aligned to show the similarity between the DMRT1 and SOX9 motifs. (**C**) NR5A2-like motif enriched in Sertoli cell ATAC-seq DARs (upper) that were not bound by SOX9 or DMRT1 and the reference NR5A2 motif from JASPAR (lower). This motif was the third ranked motif in the MEME search (*E*-value = 3.9e–084) but had the highest score when the top three motifs were used to search the JASPER_2018 non-redundant database using TOMTOM (*E*-value = 3.2e–6).

To further define how sex regulatory proteins intersect with candidate regulatory elements, we analyzed the transcription factor ChIP-seq and ATAC-seq data, looking for regulatory factor DNA binding motifs in the ovary and Sertoli cells. We first identified DNA motifs associated with FOXL2 and ESR2 binding in ovary (Figure 2A) and DMRT1 and SOX9 binding in Sertoli cells (Figure 2B). For FOXL2, ESR2 and DMRT1 the enriched motifs closely matched those previously defined by *in vitro* selection approaches and previous ChIP-seq analysis in fetal gonads (41,42). For SOX9 there were minor differences at one side of the site, with a preferred cytosine nucleotide spaced one base closer to the core consensus in the *in vivo* motif relative to the *in vitro* site. The related protein SOX2 binds distinct motifs in naked and nucleosomal DNA (43) and the SOX9 motif we identified *in vivo* more closely resembles that recognized by SOX2 in naked DNA. A cytosine at this position is also observed in the DMRT1 motif when the two motifs are aligned. The motif bound by SOX9 in Sertoli cells is very similar to the DMRT1 motif and suggests the possibility that some DNA motifs may be able to interact with either protein. Enrichment analysis also found that Sertoli-biased DARs not bound by DMRT1 or SOX9 contained a binding motif very similar to that of the nuclear hormone receptor protein NR5A2 (Figure 2C). A role for NR5A2 is Sertoli cell development has not been described. However, the close paralog NR5A1 (also called SF1 or Ad4BP) can bind this motif, plays a key role in gonadogenesis in both sexes, and activates *Sox9* expression (39), so this motif may mediate NR5A1 binding. This motif also is enriched in DARs that form during fetal gonadogenesis (6).

**Figure 3.**
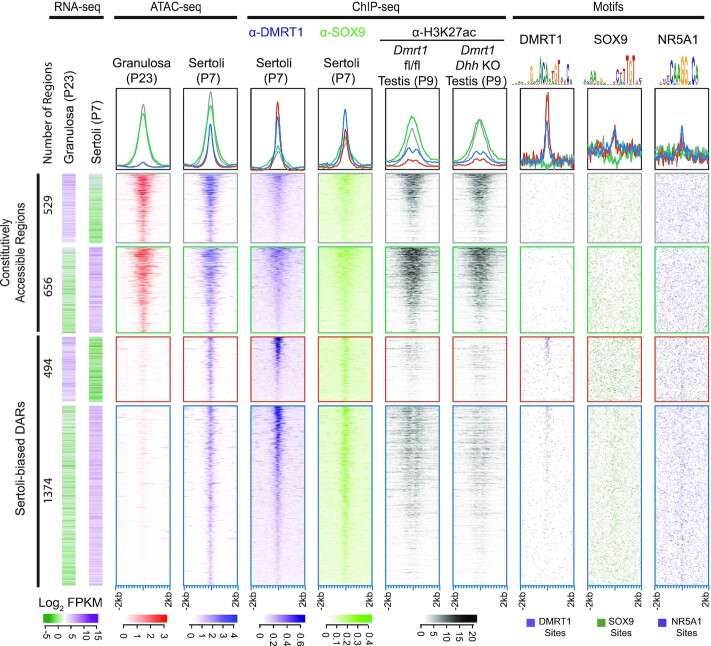
Identification of putative Sertoli regulatory elements. Expression, chromatin accessibility, binding of DMRT1 and SOX9, enrichment of H3K27ac and presence of DMRT1, SOX9, and NR5A1 DNA binding motifs in the underlying genomic sequence are shown for constitutively open regions or Sertoli-biased DARs that are located within 1kb of differentially expressed mRNAs. Heatmaps show ±2 kb from the center of the ATAC-seq peak. The constitutively accessible regions (grey and green traces) and Sertoli-biased DARs (red and blue traces) were each separated into two groups based on whether they were near a granulosa-biased transcript (grey and orange traces) or a Sertoli-biased transcript (green and blue traces). Heatmaps at bottom indicate enrichment scale for each feature. Motif heatmaps for DMRT1, SOX9 and NR5A1 show the locations of motifs that have a score ≥85%, ≥85% and ≥90% of the highest possible score for each motif respectively.

We next performed a global analysis of the Sertoli-biased DARs within 5 kb of the sex-biased genes that were defined above (Figure 3) to ask whether our observations at specific loci extend genome-wide. We categorized DARs by constitutive versus Sertoli-biased accessibility and by association with granulosa- versus Sertoli-biased coding regions. DARs from each of the four categories were then ordered based on strength of DMRT1 binding. Quantitative color-coded enrichment plots of aggregated data from each category are shown in the top row. Constitutively-accessible regions (Figure 3, grey and green boxed panels and lines in enrichment plots) were about equally associated with granulosa-biased (grey) and Sertoli-biased (green) gene bodies, and while they had some broad peaks of DMRT1 and SOX9 accumulation they lacked enrichment for DMRT1 or SOX9 binding motifs. In contrast, the Sertoli-biased DARs (red and blue boxed panels and lines in enrichment plots) had much sharper peaks of accessibility and of DMRT1 and SOX9 association, and these were enriched for DMRT1 motifs and, more modestly, for SOX9 binding motifs (Figure 3, bottom panels). The specific binding of DMRT1 and SOX9 to genomic regions within 1 kb of granulosa (red)- and Sertoli (blue)-biased genes suggests that these proteins can mediate not only transcriptional activation but also repression, as proposed previously (36). As described above, DARs near both granulosa- and Sertoli-biased genes also had some enrichment for the putative NR5A1 motif identified by enrichment analysis, suggesting a possible role in transcriptional activation and repression via these Sertoli-biased DARs. The strength of SOX9 ChIP-seq signals closely paralleled those of DMRT1 in DARs near Sertoli-biased genes, consistent with joint action of the two regulators at these DARs, while the NR5A1 motif was more prominent in regions that lacked DMRT1 and SOX9 binding. We used ChIP-seq for H3K27ac, a chromatin mark associated with active gene expression, to examine the Sertoli-biased DARs and found that it was enriched in DMRT1-bound regions. This mark was slightly decreased at DMRT1-bound DARs near Sertoli-biased transcripts in testes with *Dmrt1* conditionally deleted in Sertoli cells (compare blue enrichment plots), suggesting that DMRT1 plays a role in their transcriptional activation. While the effect of DMRT1 loss on H3K27ac enrichment in this analysis was minor, it is important to note that this ChIP-seq was performed using intact gonads and hence background from other cell types likely masked the degree of change in Sertoli cells. Collectively, the mRNA expression, chromatin accessibility, regulatory factor binding and chromatin modification data help define a set of candidate regulatory elements associated with postnatal regulation of Sertoli cell fate and suggest that some of them mediate activation and others repression by DMRT1 and/or SOX9.

### Sex-biased genome organization and gene expression

**Figure 4.**
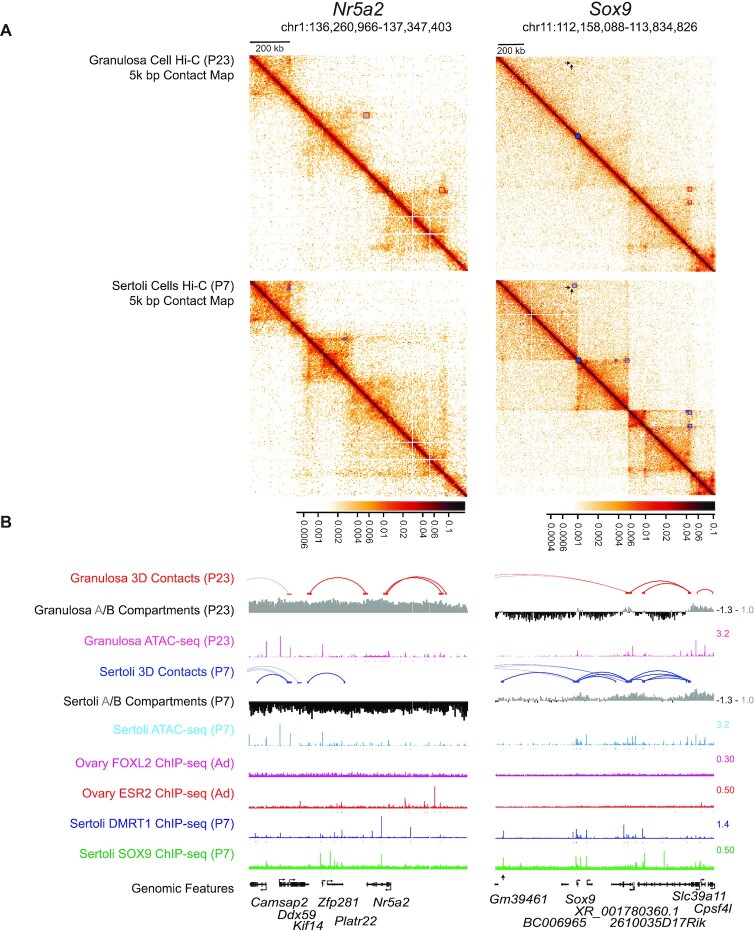
Three dimensional genome organization in female and male somatic cells. Hi-C contact maps at 5kb resolution (**A**) and one-dimensional tracks (**B**) for postnatal granulosa cells (p23) and Sertoli Cells (p7). The left hand column shows a region ∼1 Mb surrounding the granulosa expressed gene *Nr5a2*. The right hand column shows ∼1.6 Mb surrounding the Sertoli expressed gene *Sox9*. Enriched off-diagonal contacts for granulosa cells or Sertoli cells using 10kb or 25kb binning of the contact maps are shown above the diagonal as magenta or blue boxes respectively. The intensity of the 3D contacts, normalized using the iterative balancing algorithm in cooler, is shown below the contract matrices. An unobstructed view of the data is shown below the diagonal. The promoters for *Nr5a2* and *Sox9*, shown in (A) as red or blue dots on the diagonal respectively, are at domain boundaries in the permissive cell state. On the Sox9 contact map, the positions of ∼600 kb upstream enhancer located within the XYSR region (Enh13; (38)) is shown as a pair of arrowheads in the contact map and a single arrowhead below the tracks. One dimensional representations of the enriched off-diagonal contacts, A/B compartment calculated from the maps, ATAC-seq, ChIP-seq data for FOXL2, ESR2, DMRT1 and SOX9, and genomic features are shown in panel (B).

To investigate whether sexual dimorphic differences in gene expression relate to differences in the 3D organization of the genome, we performed genome-wide proximity ligation assays (Hi-C) on purified granulosa and Sertoli cells. We identified 3346 granulosa and 6657 Sertoli off-diagonal DNA interactions (indicating loops) on autosomal chromosomes that occurred more frequently than expected (false discovery rate < 0.01). We also used the Hi-C data to predict type ‘A’ and type ‘B’ chromatin compartments, which have been shown to be associated with active and inactive chromatin, respectively (44). Two chromosomal regions typifying sex-biased genomic features are shown in Figure 4. The region surrounding the granulosa-biased gene *Nr5a2* is an active type A compartment (coded grey) in granulosa cells and a repressed type B compartment (coded black) in Sertoli cells (Figure 4, left column). This region contains off-diagonal interactions in granulosa cells that overlap with granulosa-biased DARs spanning *Nr5a2* and contains multiple ESR2-bound sites. In Sertoli cells, this region contains distinct off-diagonal contacts with many Sertoli-biased DARs, a number of which are bound by DMRT1 and SOX9. In contrast, the region of the Sertoli-biased *Sox9* gene (Figure 4, right column) had predominantly type ‘B’ compartments in granulosa cells but nearly all type A compartments in Sertoli cells, consistent with its Sertoli-biased expression. A large number of off-diagonal contacts were called in Sertoli cells that contained Sertoli-biased DARs and binding sites for DMRT1 and SOX9 upstream and downstream of the *Sox9* gene. Additionally and notably, one 3D contact (black arrowheads in Figure 4A and B) connects a distal enhancer required for fetal sex determination, Enh13 (38), to the *Sox9* transcriptional start site, suggesting that this important fetal enhancer remains active postnatally. Although the causal relationship of genome organization with DARs and transcription factor binding is more difficult to infer, these data suggest a role for sex-biased genome organization in the expression differences at these loci, mediating expression at *Nr5a2* and *Sox9*. Additional examples of sex-biased chromatin association are shown in Supplemental Figure S2.

### Collaboration between DMRT1 and SOX8/9 in granulosa cell transcriptome reprogramming *in vivo*

Previous genetic analysis in the testis suggested that DMRT1 acts in concert with SOX9 to maintain male fate, because conditional deletion of both *Dmrt1* and *Sox9* causes more rapid and efficient Sertoli-to-granulosa transdifferentiation in the postnatal testis compared to deletion of *Dmrt1* alone (13). The functional relationship between DMRT1 and SOX9 has been less clear in DMRT1-induced granulosa-to-Sertoli reprogramming in the ovary. We previously found that ectopic DMRT1 expression in the ovary induces SOX9 expression in postnatal granulosa cells (17). However, we also found that ectopic DMRT1 can at least partly suppress expression of FOXL2 protein in the adult ovary even when *Sox9* and its partially redundant paralog *Sox8* are conditionally deleted in gonadal somatic cells (17). This latter result suggested that DMRT1 might have at least some sex-reprogramming activity independent of SOX8/9. However, the previous study did not examine whether DMRT1 can regulate other sex-biased genes in the absence of SOX8/9, and hence the extent to which SOX8/9 activity contributes to sexual cell fate reprogramming by DMRT1 is unknown. We therefore performed RNA-seq examining ovaries with DMRT1 ectopically expressed in somatic cells, either with or without *Sox8/9* conditionally deleted in the somatic cell lineage.

To express DMRT1 and delete *Sox8/9* we employed a previously described genetic strategy (17). In brief, we used *Sf1-Cre*, which is active from about E11.5 (26), to activate the conditional transgene *CAG-Stop^flox^-Dmrt1-eGfp* (hereafter ‘*CAG-Dmrt1*’) by deleting a ‘floxed’ set of polyadenylation sites blocking expression of *Dmrt1*, while simultaneously deleting one or two conditional alleles of *Sox9* in somatic gonadal cells of mice carrying one or two null alleles of *Sox8*. To better assess the importance of each *Sox* gene in reprogramming, we generated animals with zero, one or two functional alleles each of *Sox8* and *Sox9*, examining two to six biological replicates for each genotype (Supplemental Table S1). We then isolated and profiled mRNA from gonads of XX animals at ten weeks of age.

**Figure 5.**
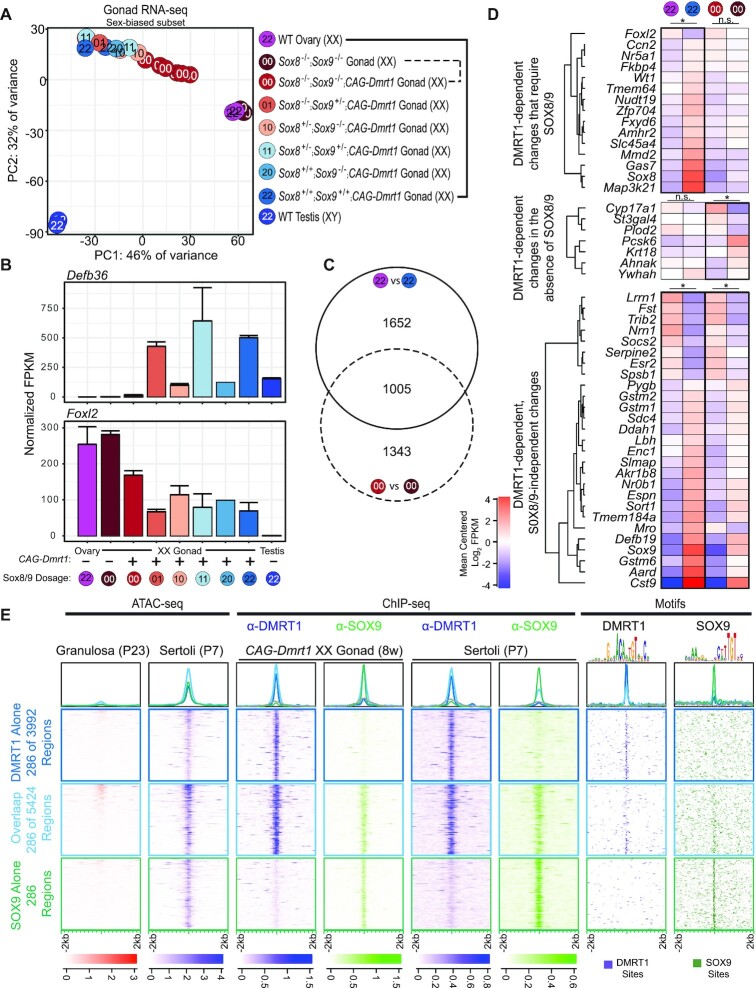
SOX8/9 contribute to sexual reprogramming by DMRT1 *in vivo*. (**A**) PCA analysis of 890 granulosa-biased genes and 872 Sertoli-biased genes in whole gonad transcriptome samples, examining Sox8/9 contribution to DMRT1 reprogramming. The variance across all 1762 genes was used to calculate the principal components. PC1 reflects sexual differentiation and PC2 mainly reflects germ cell gene expression. Colors and paired digits inside circles indicate number of intact *Sox8* and *Sox9* alleles, respectively, as indicated in key. (**B**) Dependence of *Defb36* and *Foxl2* mRNA expression on DMRT1 and SOX8/9. The genotype of the *Sox8* and *Sox9* loci are indicated below the plot and are shaded from a wild-type ovary profile on the left (magenta) to a wild-type testis on the right (navy blue) as in panel A. Error bars represent the standard error of the mean for each genotype. (**C**) Venn diagram showing proportions of genes affected by ectopic DMRT1 expression primarily when *Sox8/9* are intact (solid circle), primarily when they are missing (dashed circle), or regardless of *Sox8/9* status (overlap). (**D**) Heat map showing postnatal expression in ovary, testis, and *CAG-Dmrt1* XX gonads of mRNAs implicated in fetal somatic sex differentiation, using same genotype comparisons as panel C. An asterisk above the heatmap represents a greater than two-fold change in expression, an adjusted *P*-value less than 0.05 and mean count greater than 50 reads. n.s., not significant. (**E**) ATAC-seq analysis of isolated granulosa and Sertoli cells and ChIP-seq analysis of DMRT1 and SOX9 binding in *CAG-Dmrt1* expressing XX gonads, isolated Sertoli cells or adult testis. The locations of consensus DNA binding motifs for DMRT1 and SOX9 are also shown. Genomic regions were classified based on whether they were bound by DMRT1 (dark blue boxed panels and enrichment traces), SOX9 (green) or both DMRT1 and SOX9 (light blue). A random sample of 287 regions of the 3295 regions bound by DMRT1 alone or 2616 regions bound by both DMRT1 and SOX9 while all of the 287 regions bound by SOX9 alone are shown.

We first examined how DMRT1 and SOX8/9 affect expression of the Sertoli- and granulosa-biased mRNAs identified in Figure 1, using principal component analysis (PCA) to ask how these mRNAs respond to ectopic DMRT1 in XX gonads with zero to four functional alleles of the *Sox8* and Sox*9* genes. This analysis revealed a strong effect of *Sox8/9* dosage on reprogramming by DMRT1 in the first principal component (PC1; 46% of variance), which appears to reflect testis/ovary somatic cell fate (Figure 5A). PC2 reflected expression differences connected to variation in germ cell complement between gonads of different sexes and genotypes, and was much less affected by *Sox8/9* dosage. In XX gonads with all four copies of *Sox8/9* intact, DMRT1 was able to shift the transcriptome nearly to the control testis position in PC1. In XX gonads lacking functional alleles of *Sox8* and *Sox9*, DMRT1 was able to partially shift the transcriptome in PC1, but less than when at least one copy of *Sox8/9* was present. While a single copy of either *Sox9* or *Sox8* enhanced reprogramming by DMRT1, *Sox9* appeared more important, as presence of a functional *Sox9* allele allowed nearly full reprogramming. Importantly, deletion of both *Sox8* and *Sox9* had no effect on PC1 in XX gonads lacking DMRT1 expression, indicating that the effect of *Sox8/9* genotype on ovarian sexual cell fate is dependent on DMRT1.

The collaborative activity of DMRT1 with SOX8/9 also was apparent at the level of individual regulated mRNAs and involved both activation and repression of expression by DMRT1. For example, the strength of activation of the Sertoli-biased *Defb36* and repression of granulosa-biased *Foxl2* by DMRT1 were both proportional to the number of copies of *Sox8/9*, with *Sox9* apparently more potent than *Sox8* in regulating both genes (Figure 5B). Consistent with our previous study, DMRT1 was able to partially repress *Foxl2* in the absence of *Sox8/9* but it repressed more effectively in the presence of one or both genes, particularly *Sox9*. Among the 4000 mRNAs regulated by DMRT1 (Figure 5C), 1652 were significantly regulated only in the presence of *Sox8/9* (41%), 1343 (34%) were significantly regulated by DMRT1 only in the absence of *Sox8/*9 and 1005 (25%) were significantly regulated by DMRT1 regardless of *Sox8/9* status. We conclude that DMRT1 functionally collaborates with SOX8/9 to regulate a substantial proportion of its target genes.

### Involvement of fetal sex regulators in postnatal sexual cell fate reprogramming

We previously found that components of the fetal sex determination/differentiation network are regulated by ectopic DMRT1 expression in the postnatal ovary (17). We therefore examined expression of 462 genes related to fetal sex determination based on gene ontology, previously reported sex-biased expression in the fetal somatic gonad (45) or likely involvement in a fetal sex-regulatory circuit (46). Among these selected genes we identified 15 with DMRT1-dependent gene expression changes that required SOX8/9, seven that only responded to DMRT1 in the absence of SOX8/9 and 27 that responded to ectopic DMRT1 expression independent of SOX8/9 (Figure 5D). This result further supports the view that at least some of the transdifferentiation triggered by DMRT1 expression is mediated by SOX8 and SOX9 and involves genes that normally mediate sex differentiation in the fetal gonad.

### Similarity of binding by DMRT1 and SOX9 in sex maintenance and reprogramming

We next investigated how DMRT1 and SOX9 work together to regulate target genes, using ChIP-seq to compare their binding. We identified 95 098 and 22 548 sites bound by DMRT1 and SOX9, respectively, in eight-week XX gonads ectopically expressing DMRT1, considerably more than the 11 003 and 1360 sites bound by DMRT1 and SOX9 in wild-type Sertoli cells respectively. Of the 19 775 Sertoli-biased DARs identified in isolated Sertoli cells, 10 442 (53%) were bound by DMRT1, SOX9 or both proteins in DMRT1-expressing XX gonads. We selected sites with strong binding by either protein (peak score between the 50th and 99th percentile) that also overlapped with a previously defined Sertoli-biased DAR. To visualize this binding we generated heatmap plots and enrichment traces (Figure 5E) for randomly sampled regions stratified into three categories: regions bound by DMRT1 alone (*n* = 3992, dark blue), regions bound by DMRT1 and SOX9 (*n* = 5424, light blue) and regions bound by SOX9 alone (*n* = 286, green). DMRT1 and SOX9 were similarly bound to these regions in Sertoli cells and DMRT1-expressing XX gonads (Figure 5E). Thus, the pattern of binding by DMRT1 and SOX9 to these DARs in *CAG-Dmrt1* transgenic granulosa cells was more extensive but inclusive of the pattern observed in wild type Sertoli cells, suggesting that these regulators function similarly in female-to-male reprogramming and male sex maintenance. In addition, the joint binding of DMRT1 and SOX9 at a majority of Sertoli-biased DARs in postnatal Sertoli cells was similar to our previous observation of joint binding by these proteins in fetal Sertoli cells (41).

The binding of DMRT1 and SOX9 to many of the same DARs suggests that these two transcription factors may directly exert joint control on expression of many target genes. As expected, DMRT1 and SOX9 consensus binding motifs defined previously (Figure 2) were enriched at most sites where ChIP detected their binding (Figure 5E). This enrichment suggests that the two proteins bind mainly via their canonical consensus elements to regulate Sertoli-biased DARs. Constitutively open regions lacked binding by DMRT1 or SOX9 and did not show enrichment for either motif (not shown). The enrichment of motifs for both proteins at jointly-bound DARs suggests that they each associate directly with DNA at these loci when ectopically expressed in the ovary. Moreover, the similarity of the enriched SOX9 motif to one that is bound by SOX2 on naked but not on nucleosomal DNA, described above, suggests that SOX9 may need assistance from DMRT1 or other proteins to gain access to sites that are nucleosomal in granulosa cells.

### Ectopic DMRT1 activates SOX9 in cultured granulosa cells but ectopic SOX9 does not activate DMRT1

Ectopic DMRT1 expression in the ovary induces ectopic SOX9 expression (17,47). Our data reveal that the two proteins can act synergistically to reprogram cell fate but do not address whether SOX9 can drive sexual cell fate reprogramming without DMRT1. To better assess the potency of SOX9 in this process we sought to ectopically express it in the ovary on its own. We used a conditional transgene containing *Sox9* cDNA under control of a synthetic CAG promoter and preceded by a ‘floxed’ stop cassette (*CAG*-*mRFP1^floxed^*-*Sox9*-*eGFP*; hereafter ‘*CAG-Sox9*’) (25). SOX9 expression prior to E12.5 can cause primary sex reversal (23) so we tested two Cre drivers that are expressed later in granulosa cells, *Amhr2-Cre* (48) and *Hsd17b1-icre/ERT2* (29). Neither *Cre* transgene was able to recombine the conditional *CAG-Sox9* transgene (switching its reporter expression from RFP to eGFP) or cause ectopic SOX9 expression in the postnatal ovary (not shown). We also tried to activate *CAG-Sox9* using *Foxl2-Gfp-CreERT2*, a knock-in allele that disrupts *Foxl2* (http://www.gudmap.org/index.html). However, we found that *Foxl2* hemizygosity caused a background of ectopic SOX9 expression even in the absence of the *CAG-Sox9* transgene. Unlike the *CAG-Dmrt1* transgene, which was inserted by homologous recombination at the highly permissive *Rosa26* locus, the *CAG-Sox9* transgene was inserted at a random site by pronuclear injection, and presumably that locus is not accessible to Cre in granulosa cells *in vivo*.

**Figure 6.**
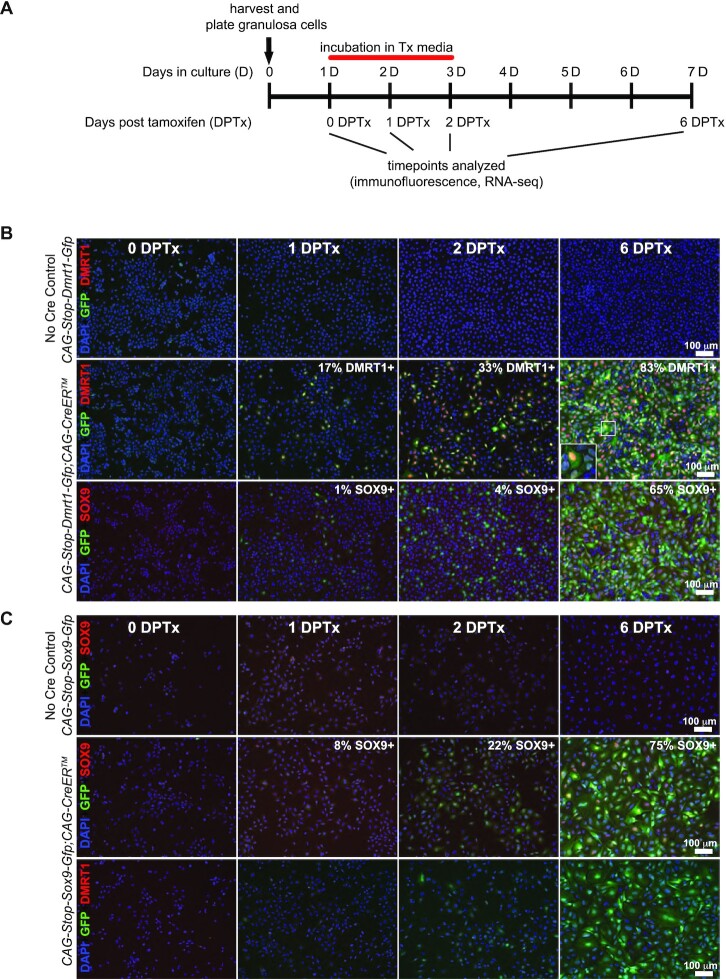
In cultured granulosa cells ectopic DMRT1 upregulates SOX9 expression but ectopic SOX9 does not upregulate DMRT1. (**A**) Diagram of culture and Tx treatment of primary granulosa cells. (**B**) Ectopic DMRT1 expression. Top row: Control cells lacking the *CAG-CreER* transgene, showing lack of GPF or DMRT1 when treated with Tx. Middle row: Granulosa cells carrying both *CAG-Dmrt1* and *CAG-CreER*, showing expression of both GFP (green) and DMRT1 (red) within one day of Tx treatment and increasing proportion of DMRT1-positive cells with time. Bottom row: DMRT1-expressing granulosa cells, showing expression of SOX9 by 1–2 DPTx and increasing proportion of SOX9-positive with time. (**C**) Top row: Control granulosa cells lacking CAG-*CreER*, showing lack of GPF or SOX9 expression after Tx treatment. Middle row: Granulosa cells carrying both *CAG-SOX9* and *CAG-CreER*, showing expression of SOX9 and GFP, SOX9-positive cells detected by one day after Tx treatment, increasing in proportion with time. Bottom row: SOX9-expressing granulosa cells, showing lack of DMRT1 expression even at 6 DPTx. Scale bars in (B) and (C) represent 100 um. Data shown are from one of three independent experiments giving very similar results.

As an alternative to *in vivo* analysis we turned to cell culture, asking whether *CAG-Sox9* can be activated in cultured primary granulosa cells. We established granulosa cell cultures from 23–29 day old ovaries as previously described (33). As a positive control we activated DMRT1 in granulosa cells from animals carrying *CAG-Dmrt1* and *CAG-CreER^TM^* (49), adding 4-hydroxy-tamoxifen (Tx) for 48 hours, starting after 24 h of culture (as diagrammed in Figure 6A). Immunofluorescence confirmed that Tx treatment efficiently activated DMRT1 and eGFP expression from the transgene (Figure 6B). DMRT1 and eGFP expression were apparent within 24 hours after tamoxifen addition and most cells (83%; *n* > 1500) were positive for both markers by six days post-tamoxifen (6 DPTx). As *in vivo*, DMRT1 expression also activated SOX9 (65% of cells were positive by 6 DPTx; *n* > 1500; Figure 6B). We next tested *CAG-Sox9*. Tamoxifen treatment of granulosa cells isolated from animals carrying *CAG-Sox9* and *CAG-CreER^TM^* activated SOX9 and GFP within 24 h, with 75% of cells SOX9-positive by 6 DPTx (*n* > 1500; Figure 6C). However, SOX9-expressing cells did not express DMRT1. From these results we conclude that ectopic DMRT1 can activate SOX9 in postnatal granulosa cells but ectopic SOX9 cannot activate DMRT1, at least under these conditions. This postnatal regulation, both *in vivo* and *in vitro*, differs from that in fetal ovaries, where ectopic DMRT1 does not activate SOX9 (17).

### Ectopic SOX9 can reprogram sex-biased gene expression *in vitro* but less robustly than DMRT1

**Figure 7.**
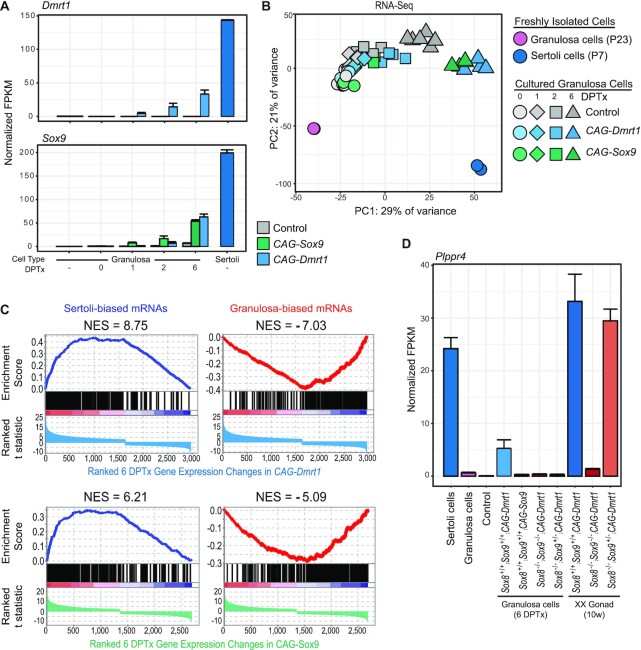
Reprogramming of sex-biased gene expression by DMRT1 and SOX9 in cultured granulosa cells. (**A**) Expression of *Dmrt1* (top) and *Sox9* (bottom) mRNAs in granulosa cells ectopically expressing DMRT1 or SOX9. Expression of *Dmrt1* and *Sox9* in freshly isolated granulosa (- DPTx) or freshly isolated Sertoli cells (dark blue bars) is also shown for comparison. Normalized FPKMs are shown for control (shaded grays), *CAG-Dmrt1* (shaded blues) and *CAG-Sox9* (shaded greens). Error bars represent the standard error of the mean at each timepoint for each of the genotypes. (**B**) PCA analysis of gene expression in cultured wild-type granulosa cells compared to granulosa cells expressing DMRT1 or SOX9. The 500 genes with the highest variance across the transcriptome were used to calculate the principal components. The symbol shapes represent the timepoint of the cultured granulosa cells as follows: 0 DPTx (circles), 1 DPTx (diamonds), 2 DPTx (squares) and 6 DPTx (triangles). (**C**) Enrichment plots showing effect of DMRT1 (top panels) and SOX9 (bottom panels) expression on Sertoli-biased (left panels) and granulosa-biased (right panels) mRNAs. The *t* statistic was used to rank the genes from the most significantly upregulated (left-hand side) to the most significantly downregulated (right-hand side) at 6 DPTx. The normalized enrichment score (NES) is shown near the curve for each panel. (**D**) *Sox*-dependence of *Plppr4* induction by DMRT1 and SOX9 in cultured granulosa cells and *in vivo*. Colors used in this panel are the same as in previous figures. Error bars represent the standard error of the mean for each genotype.

To compare the abilities of DMRT1 and SOX9 to reprogram gene expression, we performed RNA-seq on granulosa cells cultured treated with Tx as diagrammed in Figure 6A. As controls, we analyzed freshly isolated granulosa and Sertoli cells plus identically cultured granulosa cells that lacked an inducible allele or *Cre* transgene. We first examined expression of *Dmrt1* and *Sox9* mRNAs. Consistent with the immunostaining data, we found that *CAG-Dmrt1* activation resulted in upregulation of both *Dmrt1* and *Sox9* mRNA expression beginning by one day after Tx treatment (Figure 7A). In contrast, while *CAG-Sox9* produced elevated *Sox9* expression, it had no effect on *Dmrt1* mRNA expression, consistent with the protein expression data. Culturing wild-type granulosa cells under these conditions had little effect on *Dmrt1* expression but, as described previously (40) it did result in a small rise in *Sox9* mRNA expression.

Next we examined global gene expression in control granulosa cells, compared to granulosa cells expressing ectopic DMRT1 or SOX9. PCA analysis revealed that the largest principal component (PC1) was correlated with sexual cell fate, accounting for 29% of variance, and the second largest component (PC2) appeared largely due to cell culture, accounting for 21% of variance (Figure 7B). With increasing time in culture all genotypes, including control cells, trended toward the Sertoli cell position in PC1, consistent with the gradual increase in *Sox9* expression shown in Figure 7A and suggesting a partial loss of commitment to the granulosa cell fate. Expression of DMRT1 pushed granulosa cells all the way to the Sertoli position in PC1 and partially reversed the change in PC2. SOX9 expression also pushed cells toward the Sertoli position in PC1 but not as far as DMRT1. To compare the *in vitro* expression changes to *in vivo* reprogramming, we examined the expression of the Sertoli- and granulosa-biased mRNAs we had defined *in vivo* (Figure 3), using gene set enrichment analysis (GSEA). We plotted expression changes in Sertoli- and granulosa-biased mRNAs induced by DMRT1 and SOX9 six days after Tx treatment, with mRNAs ordered based on statistical significance (ranked t statistic). A higher normalized enrichment score (NES) for expression changes indicates a stronger effect on gene expression. Enrichment plots (Figure 7C) confirmed that ectopic DMRT1 or SOX9 could activate Sertoli-biased genes and repress granulosa-biased genes, and showed that ectopic DMRT1 regulated more genes of each class than ectopic SOX9 (higher NES for both Sertoli- and granulosa-biased mRNAs).

The differential and collaborative roles of DMRT1 versus SOX9 were also evident for individual Sertoli-biased genes. As an example, *Plppr4* was activated in cultured granulosa cells by expression of DMRT1 but not by expression of SOX9 (Figure 7D). However, DMRT1 was unable to activate the gene in cultured granulosa cells completely lacking *Sox8/9* or retaining just one allele of *Sox9*, indicating that both DMRT1 and SOX8/9 are required *in vitro*. *In vivo*, in *CAG-Dmrt1* expressing XX gonads, DMRT1 was unable to activate *Plppr4* expression in the absence of all *Sox8/9* alleles but, in contrast to culture, was able to activate it if even one *Sox9* allele was present. This difference illustrates that the culture system is a useful but imperfect model of *in vivo* regulation, likely due to the absence of other relevant cell types and signaling molecules. Overall, we conclude that DMRT1 and SOX9 have overlapping and distinct targets and that regulation of some targets requires both DMRT1 and SOX8/9, in culture as well as *in vivo*.

### DMRT1 mediates chromatin accessibility and allows DNA binding by SOX9

**Figure 8.**
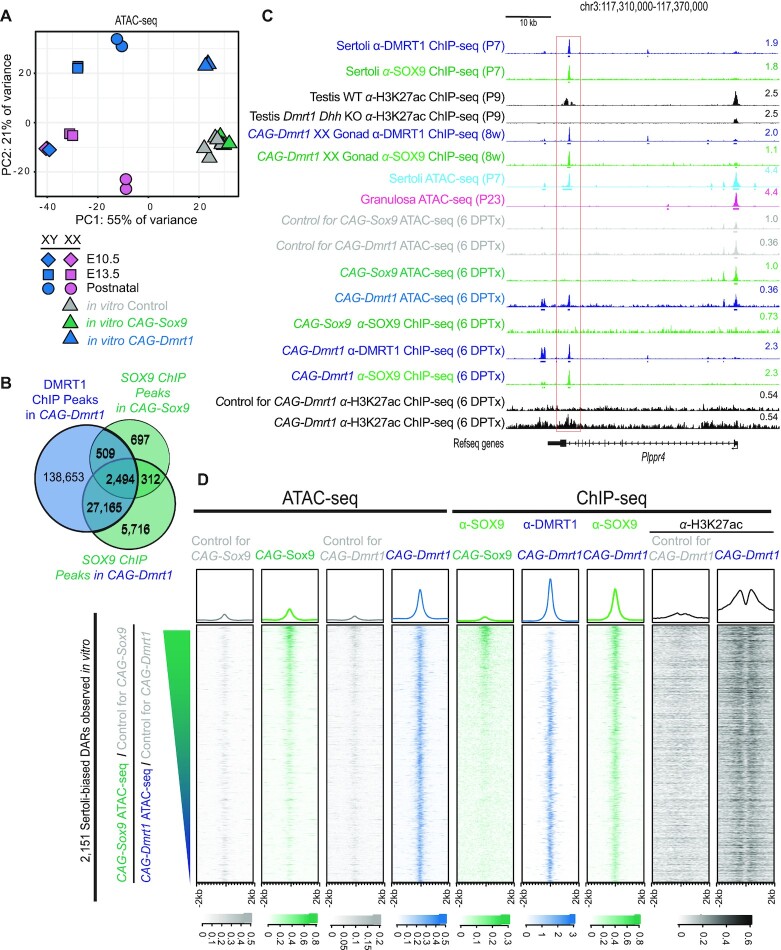
Reprogramming of chromatin accessibility by DMRT1 and SOX9 in cultured granulosa cells. (**A**) PCA analysis of ATAC-seq data from fetal and postnatal Sertoli and granulosa cells and postnatal granulosa cells ectopically expressing DMRT1 or SOX9. The 500 genomic regions with the highest variance in accessibility were used to calculate the principal components. (**B**) Venn diagram indicates the degree of overlap in ChIP-seq peaks in granulosa cells expressing *CAG-Dmrt1* or *CAG-Sox9*. DMRT1 ChIP peaks are represented by a blue circle while SOX9 ChIP peaks in *CAG-SOX9* granulosa cells (upper) and SOX9 binding in *CAG-Dmrt1* granulosa cells (lower) are represented by green circles. (**C**) ATAC-seq and ChIP-seq data at the *Plppr4* locus. The last intron of *Plppr4* contains a Sertoli-biased DAR that is bound by DMRT1 and SOX9 *in vivo* and *Dmrt1* expressing granulosa cells but not by SOX9 in *Sox9* expressing granulosa cells (red box). A DMRT1-dependent H3K27ac peak (black tracks) is observed *in vitro* and *in vivo*. For ATAC-seq and ChIP-seq data, the scale shown at right indicates the number of reads per million reads sequenced for the full height of the track. (**D**) ATAC-seq and ChIP-seq of Sertoli-biased DARs that have increased accessibility upon expression of *CAG-Sox9* or *CAG-Dmrt1* in cultured granulosa cells. DARs are sorted based on the ratio of the log_2_ fold changes in accessibility; regions with greater change in accessibility in *CAG-Sox9* expressing cells versus control cells are at the top and greater change in *CAG-Dmrt1* expressing cells versus control cells at the bottom. ChIP-seq data for SOX9, DMRT1 and H3K27ac on cultured cells is also shown. Heatmaps at bottom indicate enrichment scale for each feature. ChIP-seq and ATAC-seq experiments were performed at 6 DPTx.

Comparison of wild-type Sertoli and granulosa cells revealed many DARs with potential to control cell fate (Figure 3). To learn more about how DMRT1 and SOX9 affect DAR formation during sexual fate reprogramming, we used ATAC-seq to examine cultured granulosa cells ectopically expressing DMRT1 or SOX9. We compared published ATAC-seq data from bipotential progenitor cells and differentiating fetal pre-Sertoli and pre-granulosa cells (6) with our data from wild-type freshly isolated postnatal granulosa and Sertoli cells and from three biological replicates of postnatal cultured granulosa cells expressing ectopic DMRT1 or SOX9 and their respective controls (Figure 8A and Supplemental Table S1). In PCA analysis, the accessibility profiles of E10.5 XX and XY bipotential progenitors clustered tightly with each other, as expected since this stage precedes sexual differentiation. E13.5 pre-Sertoli cells clustered near the bipotential progenitors in PC1 but distant in PC2. This comparison suggests that PC1 is related to developmental stage while PC2 is related to sexual cell fate. In contrast to the pre-Sertoli cells, E13.5 pre-granulosa cells clustered near the E10.5 bipotential progenitors in both PC1 and PC2. This behavior is consistent with previous studies suggesting that bipotential precursors are predisposed toward the granulosa fate, based on gene expression and chromatin accessibility (4,6,45). ATAC profiles from postnatal Sertoli and granulosa cells were widely separated in PC2, consistent with their very different mRNA expression profiles. Cultured granulosa cells ectopically expressing DMRT1 were shifted most of the way to the Sertoli position in PC2, indicating that DMRT1 can induce many of the DARs that distinguish Sertoli from granulosa cells. Expression of SOX9 on its own in granulosa cells had no significant effect on the ATAC profile relative to cultured control granulosa cells. These results indicate that while SOX9 collaborates with DMRT1 to regulate sex-biased mRNA expression and in some cases is an essential partner for DMRT1, it has very little ability to induce formation of relevant male-biased accessible chromatin postnatally in the absence of DMRT1. Indeed, quantification of the data showed that ectopic DMRT1 increased accessibility at greater than ten-fold more Sertoli-biased DARs than SOX9 (2145 versus 191) in cultured granulosa cells.

The inability of SOX9 to induce Sertoli-biased DARs suggested the possibility that SOX9 might require DMRT1 in order to access some of its binding sites within these DARs. To test this prediction we examined ChIP-seq data from cultured granulosa cells ectopically expressing each protein after six days of tamoxifen induction (Figure 8B). As expected, in *CAG-Dmrt1* expressing cells DMRT1 bound many more sites than SOX9 (168 821 versus 35 687) and the majority (83%) of sites bound by SOX9 also were bound by DMRT1. SOX9 bound many more sites in cultured granulosa cells expressing DMRT1 than in those expressing only SOX9 (35 687 versus 4012) consistent with a role for DMRT1 facilitating SOX9 binding. Of the 29 659 sites bound by SOX9 and DMRT1 in *CAG-Dmrt1* expressing cells, 4353 (15%) contained close matches to the DNA sequence motifs for both SOX9 and DMRT1 as defined by the ChIP-seq experiments in Sertoli cells. A majority of these sites (77%) contained adjacent SOX9 and DMRT1 motifs, with a median distance between motifs of 81 bp, consistent with our previous description in fetal testes of a Sertoli-biased sequence signature containing motifs for both DMRT1 and SOX9 (41), while 23% had coincident DMRT1 and SOX9 motifs that align as shown in Figure 2B. Comparison of DMRT1 and SOX9 binding in Sertoli cells and when ectopically expressed in XX gonads or granulosa cells is shown in Supplemental Figure S3A, B.

As an example of a Sertoli-biased gene we examined binding of DMRT1 and SOX9 and chromatin accessibility at *Plppr4* (Figure 8C). A Sertoli-biased DAR in the last intron of *Plppr4* (boxed) bound DMRT1 and SOX9 in Sertoli cells and it was induced and bound both proteins in DMRT1-expressing XX gonads and granulosa cells. The DAR was enriched for H3K27ac in control testes and *CAG-Dmrt1* expressing cultured granulosa cells but not in testes with *Dmrt1* deleted in Sertoli cells by *Dhh-Cre*, suggesting that it is an active male regulatory element. This DAR did not form or bind SOX9 in *CAG-Sox9* expressing granulosa cells. We conclude from these results that DAR formation, SOX9 binding and H3K27ac enrichment at *Plppr4* are dependent on DMRT1. The synergy of SOX9 and DMRT1 in gene regulation and the dependence of DAR formation and SOX9 binding on DMRT1 in granulosa cells exemplified by *Plppr4* suggested two possibilities, which are not mutually exclusive. First, DMRT1 and SOX9 might bind cooperatively to some regulatory elements, with SOX9 binding requiring the presence of DMRT1 bound nearby. Second, DMRT1 might enable SOX9 binding by acting as a pioneer factor, promoting chromatin access for SOX9 at some target loci. Two additional examples of regions pioneered by DMRT1 to enable SOX9 binding as well as an example of region bound by SOX9 independently of DMRT1 are shown in Supplemental Figure S3C, D.

To ask whether DMRT1 promotes SOX9 binding more generally we investigated the occupancy of DMRT1 and SOX9 in cultured granulosa cells. We identified 2151 Sertoli-biased DARs that had increased accessibility upon expression of either CAG-*Sox9* or CAG-*Dmrt1* relative to control granulosa cells six days after tamoxifen treatment. We sorted these regions based on the ratio of the log_2_ fold-change of the accessibility induced by SOX9 and DMRT1 and examined ATAC-seq and ChIP-seq data collected for each region (Figure 8D). Regions with high accessibility in CAG-*Sox9* expressing cells were bound by SOX9 in both SOX9- and DMRT1-expressing cells. However, the ability of SOX9 to bind these regions quickly decreased as the DNA accessibility in CAG-*Sox9* expressing cells decreased. In contrast, SOX9 binding was higher in regions that had greater accessibility in CAG-*Dmrt1* expressing cells (Figure 8D). Thus, SOX9 binding to these Sertoli-biased DARs was strongly DMRT1-dependent, consistent with DMRT1 playing an essential role in opening chromatin to allow access by SOX9. DMRT1 and SOX9 binding to these DARs also was associated with H3K27ac enrichment in *CAG-Dm*rt1 expressing granulosa cells relative to control cells (Figure 8D, right two columns), suggesting that these regions may serve as DMRT1-responsive transcriptional regulatory elements for Sertoli cell fate specification.

## DISCUSSION

DMRT1 is required continuously in the pubertal and adult testis to suppress expression of FOXL2, ESR2 and other female regulators that can otherwise drive male-to-female transdifferentiation (10). As such, DMRT1 anchors an active regulatory network that safeguards male fate in the postnatal XY gonad. DMRT1 also can impose male fate, reprogramming granulosa cells from female to male when ectopically expressed in the pubertal or adult ovary (17). In the testis, we found previously that combined loss of *Dmrt1* and *Sox8/9* causes more severe sexual transdifferentiation than loss of *Dmrt1* or *Sox8/9* alone, indicating that DMRT1 maintains male sexual cell fate in partnership with SOX8/9 (13). In this study we found that DMRT1 also acts with SOX8/9 in female-to-male reprogramming and we investigated how these transcription factors jointly reprogram sexual cell fate *in vivo* and in primary cell culture. We found that in Sertoli cells DMRT1 and SOX9 bind many of the same sex-specifically accessible chromatin sites, that DMRT1 can render postnatal granulosa cell chromatin accessible for SOX9 binding, and that both transcription factors are required for the full regulation of many sex-biased mRNAs.

To find relevant genes and regulatory regions, we first identified mRNAs that are differentially expressed in postnatal Sertoli and granulosa cells and differentially accessible chromatin regions (DARs) that are biased to each cell type. Many of the sex-biased DARs were associated with genes with sex-biased expression. The sex-biased genes linked to granulosa-biased DARs had primarily granulosa-biased expression, as expected if these DARs mainly mediate positive regulation of female fate. Similarly, sex-biased genes associated with Sertoli-biased DARs were primarily Sertoli-biased, suggesting mainly positive regulation of male fate. However, association of female- and male-biased DARs with genes that had opposite expression bias suggests that some of these DARs also mediate negative regulation of sexual cell fate. Such mixed regulation also occurs during fetal male development. For example, in fetal XY gonads SOX9 is required to activate testis-biased genes, while Polycomb Repressive Complex 1 protein CBX2 is required to repress ovarian gene expression (50).

In Sertoli cells about one-quarter of the Sertoli-biased DARs were bound by DMRT1, SOX9 or both, suggesting that they are controlled directly by these key sex regulators. Moreover, loss of DMRT1 reduced enrichment of the ‘active’ chromatin mark H3K27ac at many Sertoli-biased DARs. Based on PCA analysis, the constellation of postnatal DARs relevant to Sertoli identity that is induced by DMRT1 in granulosa cells overlaps that in differentiating fetal pre-Sertoli cells (Figure 8A) (6,38)). This overlap suggests that postnatal sex reprogramming is mechanistically similar to fetal sex differentiation, as also suggested by our previous single-cell transcriptome analysis (17).

We observed two general patterns of accessibility and regulatory factor binding among sex-biased DARs. At some genes, such as *Sox9*, binding by DMRT1 causes a change in DNA accessibility that may help promote 3D chromatin reorganization from a repressed compartment to an active compartment. At other genes, such as *Esr2*, changes in ‘A/B’ compartments were not observed, yet sex-biased DARs formed and were bound by key regulators in each sex. In the case of *Esr2*, a Sertoli-biased DAR was bound by DMRT1 and a granulosa-biased DAR was bound by FOXL2 and ESR2. The convergence of male and female regulation at nearby DARs is consistent with the idea that *Esr2* acts as a key ‘pivot point’ for control of sexual cell fate, a model also supported by the postnatal female-to-male transdifferentiation that results from deletion of *Esr2* and its close paralog *Esr1* in the ovary (8,9). Indeed, DMRT1 expression can silence *Esr2* expression and FOXL2 can activate it via the granulosa-biased DAR (17,40). In addition to the binding of FOXL2 and ESR2 at the *Esr2* locus, we detected ESR2 binding near the *Foxl2* coding region, consistent with feed-forward regulation of female sex maintenance by these two transcription factors.

In XX gonads ectopically expressing DMRT1, collaboration with SOX8/9 was evident both globally, at the level of the transcriptome, and in control of individual genes including *Foxl2, Esr2*, and other sex regulators. About 40% of genes that responded to ectopic DMRT1 expression did not require SOX8/9 for their response (1652/4000 genes), but the rest required at least one functional *Sox8/9* allele in order to respond to DMRT1. Our results suggest that transdifferentiation results both from the joint control of a shared target gene set and from the additive effects of distinct target gene sets controlled separately by DMRT1 or SOX8/9. ChIP-seq analysis indicated that many regulatory elements are jointly controlled by these regulators. In this regard the relationship of DMRT1 and SOX9 appears similar to that in the fetal gonad, where we previously showed that the two proteins bind near one another at many sites (41). In reprogramming, however, ChIP-seq suggested that DMRT1 plays the dominant role: of the Sertoli-biased DARs bound by DMRT1 and/or SOX9 in the ovary, only 4% were bound by SOX9 and not DMRT1. Thus, DMRT1 not only activates SOX9 expression but is joined by SOX9 at most of its Sertoli-biased putative regulatory sites.

In granulosa cell culture, we found that ectopic DMRT1 expression activated *Sox8/9* as it does *in vivo* in the postnatal ovary (here and (17)). However, ectopic SOX9 did not activate DMRT1, which allowed its effects on chromatin and gene expression to be assessed in the absence of DMRT1. Similar to the *in vivo* analysis, in culture we found that SOX9 and DMRT1 both contribute to sexual reprogramming of gene expression, with the strongest reprogramming resulting when both proteins are expressed. The non-reciprocal regulation of DMRT1 and SOX8/9 suggests that they do not function in a feed-forward loop in reprogramming, at least in cultured granulosa cells. A caveat, however, is that neither *Dmrt1* nor *Sox9* was expressed ectopically in granulosa cells at levels as high as their endogenous levels in Sertoli cells (Figure 7A).

**Figure 9.**
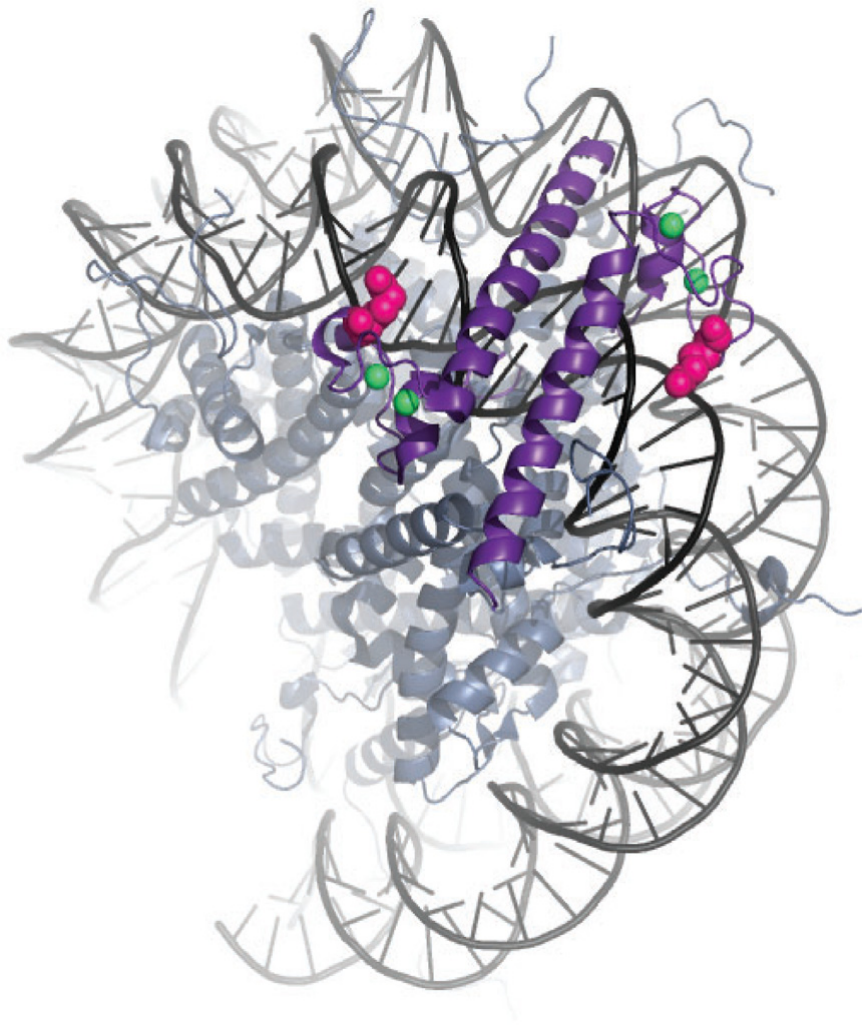
Model of DMRT1 bound to nucleosomal DNA. Molecular model of DMRT1 DM domain structure 4YJ0 (16) bound to DNA in nucleosome 1KX5 (65). Image was made by aligning the C1’ carbons of the nucleotides G9,A12 of strand D and T14,C17 of strand E from DMRT1 with T-38,G-35 from strand J and C35,A38 of strand I of the nucleosome using the pair_fit function in MacPyMOL. Zinc atoms are shown as green spheres and a space-filling model for arginine 72, which inserts into the minor groove, is shown in pink.

As *in vivo*, ChIP-seq in cultured cells indicated that many sites were bound jointly by DMRT1 and SOX9 but SOX9 was unable to bind most of these jointly occupied sites when it was expressed without DMRT1. This dependence by SOX9 on DMRT1 for DNA association suggests at least two possibilities. One possibility, given the sequence similarity of DMRT1 and SOX9 motifs that are enriched in co-bound DARs (Figure 2), is that DMRT1 and SOX9 might interact with DNA concurrently, with DMRT1 and SOX9 forming a heteromeric complex to stably bind to sites that SOX9 cannot bind alone. Structural studies suggest this mechanism is highly unlikely. The DM domain of DMRT1 binds to a widened major groove and employs an arginine side chain to read the shape of the minor groove (16). This binding is unlikely to be compatible with concurrent minor groove binding by the HMG domain of SOX9. HMG domains bind in a widened minor groove and induce bending of the helical axis of the DNA (51). Instead, the mechanism we favor, since DMRT1 can induce Sertoli-biased DARs in granulosa cells, is that DMRT1 acts as a pioneer factor, activating *Sox9* expression and promoting chromatin accessibility at regions that are then bound by SOX9 and potentially other sex regulators. It is possible that DMRT1 and SOX9 bind to the same DNA element sequentially at some sites, with DMRT1 binding first. The SOX9 motif detected in ChIP-seq was similar to a SOX2 motif involved in binding of naked DNA. This similarity further suggests that SOX9 may require DMRT1 to provide access to its sites if they are incorporated in nucleosomes. Expression of DMRT1 results in many DMRT1-bound DARs and molecular modeling (Figure 9) suggests that DMRT1 can interact sequence-specifically with nucleosomal DNA, as would be required of a pioneer factor. Biochemical experiments will be needed to confirm the ability of DMRT1 to open chromatin and investigate the mechanism by which it does so.

Our study highlights similarities and differences between fetal and postnatal sexual fate regulation. DMRT1 expression in postnatal granulosa cells induces formation of DARs and gene expression changes that overlap with those normally found in developing pre-Sertoli cells and DMRT1 binds near SOX9 at many sites postnatally and fetally. We also found key differences. For example, SOX9 alone cannot induce many Sertoli-biased DARs in cultured postnatal granulosa cells and it cannot bind many sex-biased DARs in culture or *in vivo* without help from DMRT1. In contrast, in the fetal XX gonad, ectopic SOX9 but not DMRT1 is sufficient for determination of male sexual fate (31,34,52,53). Perhaps, therefore, in the fetal gonad SOX9 can access its crucial sex regulatory sites without help from DMRT1. The molecular basis of this difference is unclear. One possibility is that the expression of DMRT1 in fetal gonads of both sexes prior to sexual differentiation enables SOX9 to access its key regulatory sites. Another possibility is that the fetal chromatin is more generally accessible than postnatal chromatin at key regulatory sites prior to the sex-biased ATAC sensitivity that develops at many sites later in fetal and postnatal development. This is particularly likely in the bipotential progenitor cells, which have not yet begun to acquire sex-biased chromatin accessibility. Another important difference that is not yet explained is why DMRT1 can activate SOX9 postnatally but not in the fetal gonad. It is possible that protective mechanisms prevent inappropriate SOX9 expression during early granulosa development. Previous observations that loss of FOXL2 activates SOX9 in the postnatal but not the fetal gonad (7,54) are consistent with such protection. The existence of such a fetal block remains speculative, but a miRNA that represses *Sox9* in fetal granulosa cells has been proposed to play such a role (55). This regulation is unlikely to fully explain the difference in reprogrammability between fetal and postnatal ovaries, however: DMRT1 can substantially reprogram sexual cell fate in the postnatal ovary even in the absence of SOX8/9 but ectopic DMRT1 expression has little or no effect on fetal granulosa cell development (17).

In summary, we have found that DMRT1 collaborates with SOX9 to reprogram sexual cell fate, overriding female fate and imposing male fate in postnatal granulosa cells, and does so in part by establishing sex-biased accessible regions that are bound and regulated jointly with SOX9. DMRT1 and SOX9 are conserved across vertebrate sexual development, as are FOXL2 and ESR (56–63), so we speculate that the transcriptional networks controlling sexual cell fate in the gonad may be regulated similarly in other vertebrate species. In addition, since DMRT proteins are conserved across metazoans and regulate sexual development in nearly all species examined to date, our findings may have relevance for establishment, maintenance, and reprogramming of sexual cell fate in other phyla.

## DATA AVAILABILITY

Published data used in this paper are available through the Gene Expression Omnibus with accession numbers GSE64960 (Lindeman *et al.* 2015) and GSE118755 (Garcia-Moreno et. al, 2019). Data generated for this study are available under the accession number GSE154484 and at the 4D nucleome data portal under the accession numbers 4DNES265ETYQ and 4DNESSS7VU57.

## Supplementary Material

gkab448_Supplemental_Files

## Notes

Present address: Kellie Agrimson, Dept. of Biology, St. Catherine University, Saint Paul, MN 55105, USA.

Present address: Rachel Gewiss, School of Molecular Biosciences, College of Veterinary Medicine, Washington State University, Pullman, WA 99164, USA.

Present address: Robin Lindeman, Beckman Coulter Inc., Chaska, MN 55318, USA.

## References

[1] Albrecht K.H. , Eicher E.M. Evidence that Sry is expressed in pre-Sertoli cells and Sertoli and granulosa cells have a common precursor. Dev. Biol. 2001; 240:92–107. 11784049 10.1006/dbio.2001.0438

[2] Lin Y.T. , Capel B. Cell fate commitment during mammalian sex determination. Curr. Opin. Genet. Dev. 2015; 32:144–152. 25841206 10.1016/j.gde.2015.03.003 PMC4470863

[3] Svingen T. , Koopman P. Building the mammalian testis: origins, differentiation, and assembly of the component cell populations. Genes Dev. 2013; 27:2409–2426. 24240231 10.1101/gad.228080.113 PMC3841730

[4] Jameson S.A. , Natarajan A., Cool J., DeFalco T., Maatouk D.M., Mork L., Munger S.C., Capel B. Temporal transcriptional profiling of somatic and germ cells reveals biased lineage priming of sexual fate in the fetal mouse gonad. PLoS Genet. 2012; 8:e1002575. 22438826 10.1371/journal.pgen.1002575 PMC3305395

[5] Stevant I. , Kuhne F., Greenfield A., Chaboissier M.C., Dermitzakis E.T., Nef S. Dissecting cell lineage specification and sex fate determination in gonadal somatic cells using single-cell transcriptomics. Cell Rep. 2019; 26:3272–3283. 30893600 10.1016/j.celrep.2019.02.069

[6] Garcia-Moreno S.A. , Futtner C.R., Salamone I.M., Gonen N., Lovell-Badge R., Maatouk D.M. Gonadal supporting cells acquire sex-specific chromatin landscapes during mammalian sex determination. Dev. Biol. 2019; 446:168–179. 30594505 10.1016/j.ydbio.2018.12.023 PMC6368449

[7] Uhlenhaut N.H. , Jakob S., Anlag K., Eisenberger T., Sekido R., Kress J., Treier A.C., Klugmann C., Klasen C., Holter N.I. et al. Somatic sex reprogramming of adult ovaries to testes by FOXL2 ablation. Cell. 2009; 139:1130–1142. 20005806 10.1016/j.cell.2009.11.021

[8] Couse J.F. , Hewitt S.C., Bunch D.O., Sar M., Walker V.R., Davis B.J., Korach K.S. Postnatal sex reversal of the ovaries in mice lacking estrogen receptors alpha and beta. Science. 1999; 286:2328–2331. 10600740 10.1126/science.286.5448.2328

[9] Dupont S. , Dennefeld C., Krust A., Chambon P., Mark M. Expression of Sox9 in granulosa cells lacking the estrogen receptors, ERalpha and ERbeta. Dev. Dyn. 2003; 226:103–106. 12508230 10.1002/dvdy.10202

[10] Matson C.K. , Murphy M.W., Sarver A.L., Griswold M.D., Bardwell V.J., Zarkower D. DMRT1 prevents female reprogramming in the postnatal mammalian testis. Nature. 2011; 476:101–104. 21775990 10.1038/nature10239 PMC3150961

[11] Georg I. , Barrionuevo F., Wiech T., Scherer G. Sox9 and Sox8 are required for basal lamina integrity of testis cords and for suppression of FOXL2 during embryonic testis development in mice. Biol. Reprod. 2012; 87:99. 22837482 10.1095/biolreprod.112.101907

[12] Barrionuevo F.J. , Hurtado A., Kim G.J., Real F.M., Bakkali M., Kopp J.L., Sander M., Scherer G., Burgos M., Jimenez R. Sox9 and Sox8 protect the adult testis from male-to-female genetic reprogramming and complete degeneration. Elife. 2016; 5:e15635. 27328324 10.7554/eLife.15635 PMC4945155

[13] Minkina A. , Matson C.K., Lindeman R.E., Ghyselinck N.B., Bardwell V.J., Zarkower D. DMRT1 protects male gonadal cells from retinoid-dependent sexual transdifferentiation. Dev. Cell. 2014; 29:511–520. 24856513 10.1016/j.devcel.2014.04.017 PMC4105363

[14] Raymond C.S. , Shamu C.E., Shen M.M., Seifert K.J., Hirsch B., Hodgkin J., Zarkower D. Evidence for evolutionary conservation of sex-determining genes. Nature. 1998; 391:691–695. 9490411 10.1038/35618

[15] Matson C.K. , Zarkower D. Sex and the singular DM domain: insights into sexual regulation, evolution and plasticity. Nat. Rev. Genet. 2012; 13:163–174. 22310892 10.1038/nrg3161 PMC3595575

[16] Murphy M.W. , Lee J.K., Rojo S., Gearhart M.D., Kurahashi K., Banerjee S., Loeuille G.A., Bashamboo A., McElreavey K., Zarkower D. et al. An ancient protein-DNA interaction underlying metazoan sex determination. Nat. Struct. Mol. Biol. 2015; 22:442–451. 26005864 10.1038/nsmb.3032 PMC4476070

[17] Lindeman R.E. , Gearhart M.D., Minkina A., Krentz A.D., Bardwell V.J., Zarkower D. Sexual cell-fate reprogramming in the ovary by DMRT1. Curr. Biol. 2015; 25:764–771. 25683803 10.1016/j.cub.2015.01.034 PMC4366330

[18] Guo C. , Morris S.A. Engineering cell identity: establishing new gene regulatory and chromatin landscapes. Curr. Opin. Genet. Dev. 2017; 46:50–57. 28667865 10.1016/j.gde.2017.06.011

[19] Davis R.L. , Weintraub H., Lassar A.B. Expression of a single transfected cDNA converts fibroblasts to myoblasts. Cell. 1987; 51:987–1000. 3690668 10.1016/0092-8674(87)90585-x

[20] Ieda M. , Fu J.D., Delgado-Olguin P., Vedantham V., Hayashi Y., Bruneau B.G., Srivastava D. Direct reprogramming of fibroblasts into functional cardiomyocytes by defined factors. Cell. 2010; 142:375–386. 20691899 10.1016/j.cell.2010.07.002 PMC2919844

[21] Vierbuchen T. , Ostermeier A., Pang Z.P., Kokubu Y., Sudhof T.C., Wernig M. Direct conversion of fibroblasts to functional neurons by defined factors. Nature. 2010; 463:1035–1041. 20107439 10.1038/nature08797 PMC2829121

[22] Zhou Q. , Brown J., Kanarek A., Rajagopal J., Melton D.A. In vivo reprogramming of adult pancreatic exocrine cells to beta-cells. Nature. 2008; 455:627–632. 18754011 10.1038/nature07314 PMC9011918

[23] Vidal V.P. , Chaboissier M.C., de Rooij D.G., Schedl A. Sox9 induces testis development in XX transgenic mice. Nat. Genet. 2001; 28:216–217. 11431689 10.1038/90046

[24] Lei N. , Hornbaker K.I., Rice D.A., Karpova T., Agbor V.A., Heckert L.L. Sex-specific differences in mouse DMRT1 expression are both cell type- and stage-dependent during gonad development. Biol. Reprod. 2007; 77:466–475. 17567962 10.1095/biolreprod.106.058784 PMC2580730

[25] Kim Y. , Murao H., Yamamoto K., Deng J.M., Behringer R.R., Nakamura T., Akiyama H. Generation of transgenic mice for conditional overexpression of Sox9. J. Bone Miner. Metab. 2011; 29:123–129. 20676705 10.1007/s00774-010-0206-z PMC3977853

[26] Bingham N.C. , Verma-Kurvari S., Parada L.F., Parker K.L. Development of a steroidogenic factor 1/Cre transgenic mouse line. Genesis. 2006; 44:419–424. 16937416 10.1002/dvg.20231

[27] Lindeboom F. , Gillemans N., Karis A., Jaegle M., Meijer D., Grosveld F., Philipsen S. A tissue-specific knockout reveals that Gata1 is not essential for Sertoli cell function in the mouse. Nucleic Acids Res. 2003; 31:5405–5412. 12954777 10.1093/nar/gkg723 PMC203309

[28] Ruzankina Y. , Pinzon-Guzman C., Asare A., Ong T., Pontano L., Cotsarelis G., Zediak V.P., Velez M., Bhandoola A., Brown E.J. Deletion of the developmentally essential gene ATR in adult mice leads to age-related phenotypes and stem cell loss. Cell Stem Cell. 2007; 1:113–126. 18371340 10.1016/j.stem.2007.03.002 PMC2920603

[29] Grabner B. , Blaas L., Musteanu M., Hoffmann T., Birbach A., Eferl R., Casanova E. A mouse tool for conditional mutagenesis in ovarian granulosa cells. Genesis. 2010; 48:612–617. 20715176 10.1002/dvg.20664

[30] Bi W. , Huang W., Whitworth D.J., Deng J.M., Zhang Z., Behringer R.R., de Crombrugghe B. Haploinsufficiency of Sox9 results in defective cartilage primordia and premature skeletal mineralization. Proc. Natl. Acad. Sci. U.S.A. 2001; 98:6698–6703. 11371614 10.1073/pnas.111092198 PMC34415

[31] Chaboissier M.C. , Kobayashi A., Vidal V.I., Lutzkendorf S., van de Kant H.J., Wegner M., de Rooij D.G., Behringer R.R., Schedl A. Functional analysis of Sox8 and Sox9 during sex determination in the mouse. Development. 2004; 131:1891–1901. 15056615 10.1242/dev.01087

[32] Kim S. , Bardwell V.J., Zarkower D. Cell type-autonomous and non-autonomous requirements for Dmrt1 in postnatal testis differentiation. Dev. Biol. 2007; 307:314–327. 17540358 10.1016/j.ydbio.2007.04.046 PMC1995593

[33] Gong X. , McGee E.A. Smad3 is required for normal follicular follicle-stimulating hormone responsiveness in the mouse. Biol. Reprod. 2009; 81:730–738. 19535790 10.1095/biolreprod.108.070086 PMC6058743

[34] Raymond C.S. , Murphy M.W., O'Sullivan M.G., Bardwell V.J., Zarkower D. Dmrt1, a gene related to worm and fly sexual regulators, is required for mammalian testis differentiation. Genes Dev. 2000; 14:2587–2595. 11040213 10.1101/gad.834100 PMC316999

[35] Notarnicola C. , Malki S., Berta P., Poulat F., Boizet-Bonhoure B. Transient expression of SOX9 protein during follicular development in the adult mouse ovary. Gene Expr. Patterns. 2006; 6:695–702. 16488195 10.1016/j.modgep.2006.01.001

[36] Murphy M.W. , Sarver A.L., Rice D., Hatzi K., Ye K., Melnick A., Heckert L.L., Zarkower D., Bardwell V.J. Genome-wide analysis of DNA binding and transcriptional regulation by the mammalian Doublesex homolog DMRT1 in the juvenile testis. Proc. Natl. Acad. Sci. U.S.A. 2010; 107:13360–13365. 20616082 10.1073/pnas.1006243107 PMC2922116

[37] Buenrostro J.D. , Wu B., Chang H.Y., Greenleaf W.J. ATAC-seq: a method for assaying chromatin accessibility genome-wide. Curr Protoc Mol Biol. 2015; 109:21.29.1–21.29.9. 10.1002/0471142727.mb2129s109 PMC4374986 25559105

[38] Gonen N. , Futtner C.R., Wood S., Garcia-Moreno S.A., Salamone I.M., Samson S.C., Sekido R., Poulat F., Maatouk D.M., Lovell-Badge R. Sex reversal following deletion of a single distal enhancer of Sox9. Science. 2018; 360:1469–1473. 29903884 10.1126/science.aas9408 PMC6034650

[39] Sekido R. , Lovell-Badge R. Sex determination involves synergistic action of SRY and SF1 on a specific Sox9 enhancer. Nature. 2008; 453:930–934. 18454134 10.1038/nature06944

[40] Georges A. , L’Hote D., Todeschini A.L., Auguste A., Legois B., Zider A., Veitia R.A. The transcription factor FOXL2 mobilizes estrogen signaling to maintain the identity of ovarian granulosa cells. Elife. 2014; 3:e04207. 10.7554/eLife.04207 PMC4356143 25369636

[41] Rahmoun M. , Lavery R., Laurent-Chaballier S., Bellora N., Philip G.K., Rossitto M., Symon A., Pailhoux E., Cammas F., Chung J. et al. In mammalian foetal testes, SOX9 regulates expression of its target genes by binding to genomic regions with conserved signatures. Nucleic Acids Res. 2017; 45:7191–7211. 28472341 10.1093/nar/gkx328 PMC5499551

[42] Krentz A.D. , Murphy M.W., Zhang T., Sarver A.L., Jain S., Griswold M.D., Bardwell V.J., Zarkower D. Interaction between DMRT1 function and genetic background modulates signaling and pluripotency to control tumor susceptibility in the fetal germ line. Dev. Biol. 2013; 377:67–78. 23473982 10.1016/j.ydbio.2013.02.014 PMC3630265

[43] Soufi A. , Garcia M.F., Jaroszewicz A., Osman N., Pellegrini M., Zaret K.S. Pioneer transcription factors target partial DNA motifs on nucleosomes to initiate reprogramming. Cell. 2015; 161:555–568. 25892221 10.1016/j.cell.2015.03.017 PMC4409934

[44] Lieberman-Aiden E. , van Berkum N.L., Williams L., Imakaev M., Ragoczy T., Telling A., Amit I., Lajoie B.R., Sabo P.J., Dorschner M.O. et al. Comprehensive mapping of long-range interactions reveals folding principles of the human genome. Science. 2009; 326:289–293. 19815776 10.1126/science.1181369 PMC2858594

[45] Munger S.C. , Natarajan A., Looger L.L., Ohler U., Capel B. Fine time course expression analysis identifies cascades of activation and repression and maps a putative regulator of mammalian sex determination. PLos Genet. 2013; 9:e1003630. 23874228 10.1371/journal.pgen.1003630 PMC3708841

[46] Capel B. Vertebrate sex determination: evolutionary plasticity of a fundamental switch. Nat. Rev. Genet. 2017; 18:675–689. 28804140 10.1038/nrg.2017.60

[47] Zhao L. , Svingen T., Ng E.T., Koopman P. Female-to-male sex reversal in mice caused by transgenic overexpression of Dmrt1. Development. 2015; 142:1083–1088. 25725066 10.1242/dev.122184

[48] Jorgez C.J. , Klysik M., Jamin S.P., Behringer R.R., Matzuk M.M. Granulosa cell-specific inactivation of follistatin causes female fertility defects. Mol. Endocrinol. 2004; 18:953–967. 14701941 10.1210/me.2003-0301

[49] Hayashi S. , McMahon A.P. Efficient recombination in diverse tissues by a tamoxifen-inducible form of Cre: a tool for temporally regulated gene activation/inactivation in the mouse. Dev. Biol. 2002; 244:305–318. 11944939 10.1006/dbio.2002.0597

[50] Garcia-Moreno S.A. , Lin Y.T., Futtner C.R., Salamone I.M., Capel B., Maatouk D.M. CBX2 is required to stabilize the testis pathway by repressing Wnt signaling. PLoS Genet. 2019; 15:e1007895. 31116734 10.1371/journal.pgen.1007895 PMC6548405

[51] McDowall S. , Argentaro A., Ranganathan S., Weller P., Mertin S., Mansour S., Tolmie J., Harley V. Functional and structural studies of wild type SOX9 and mutations causing campomelic dysplasia. J. Biol. Chem. 1999; 274:24023–24030. 10446171 10.1074/jbc.274.34.24023

[52] Barrionuevo F. , Bagheri-Fam S., Klattig J., Kist R., Taketo M.M., Englert C., Scherer G. Homozygous inactivation of Sox9 causes complete XY sex reversal in mice. Biol. Reprod. 2006; 74:195–201. 16207837 10.1095/biolreprod.105.045930

[53] Lavery R. , Lardenois A., Ranc-Jianmotamedi F., Pauper E., Gregoire E.P., Vigier C., Moreilhon C., Primig M., Chaboissier M.C. XY Sox9 embryonic loss-of-function mouse mutants show complete sex reversal and produce partially fertile XY oocytes. Dev. Biol. 2011; 354:111–122. 21466799 10.1016/j.ydbio.2011.03.029

[54] Veitia R.A. FOXL2 versus SOX9: a lifelong “battle of the sexes. Bioessays. 2010; 32:375–380. 20414895 10.1002/bies.200900193

[55] Real F.M. , Sekido R., Lupianez D.G., Lovell-Badge R., Jimenez R., Burgos M. A microRNA (mmu-miR-124) prevents Sox9 expression in developing mouse ovarian cells. Biol. Reprod. 2013; 89:78. 23946534 10.1095/biolreprod.113.110957

[56] Baron D. , Batista F., Chaffaux S., Cocquet J., Cotinot C., Cribiu E., De Baere E., Guiguen Y., Jaubert F., Pailhoux E. et al. Foxl2 gene and the development of the ovary: a story about goat, mouse, fish and woman. Reprod. Nutr. Dev. 2005; 45:377–382. 15982462 10.1051/rnd:2005028

[57] Kossack M.E. , Draper B.W. Genetic regulation of sex determination and maintenance in zebrafish (Danio rerio). Curr. Top. Dev. Biol. 2019; 134:119–149. 30999973 10.1016/bs.ctdb.2019.02.004 PMC6894417

[58] Smith C.A. , Roeszler K.N., Ohnesorg T., Cummins D.M., Farlie P.G., Doran T.J., Sinclair A.H. The avian Z-linked gene DMRT1 is required for male sex determination in the chicken. Nature. 2009; 461:267–271. 19710650 10.1038/nature08298

[59] Matsuda M. , Nagahama Y., Shinomiya A., Sato T., Matsuda C., Kobayashi T., Morrey C.E., Shibata N., Asakawa S., Shimizu N. et al. DMY is a Y-specific DM-domain gene required for male development in the medaka fish. Nature. 2002; 417:559–563. 12037570 10.1038/nature751

[60] Masuyama H. , Yamada M., Kamei Y., Fujiwara-Ishikawa T., Todo T., Nagahama Y., Matsuda M. Dmrt1 mutation causes a male-to-female sex reversal after the sex determination by Dmy in the medaka. Chromosome Res. 2012; 20:163–176. 22187367 10.1007/s10577-011-9264-x

[61] Lu H. , Cui Y., Jiang L., Ge W. Functional analysis of nuclear estrogen receptors in zebrafish reproduction by genome editing approach. Endocrinology. 2017; 158:2292–2308. 28398516 10.1210/en.2017-00215

[62] Kent J. , Wheatley S.C., Andrews J.E., Sinclair A.H., Koopman P. A male-specific role for SOX9 in vertebrate sex determination. Development. 1996; 122:2813–2822. 8787755 10.1242/dev.122.9.2813

[63] Loffler K.A. , Zarkower D., Koopman P. Etiology of ovarian failure in blepharophimosis ptosis epicanthus inversus syndrome: FOXL2 is a conserved, early-acting gene in vertebrate ovarian development. Endocrinology. 2003; 144:3237–3243. 12810580 10.1210/en.2002-0095

[64] Murphy M.W. , Zarkower D., Bardwell V.J. Vertebrate DM domain proteins bind similar DNA sequences and can heterodimerize on DNA. BMC Mol. Biol. 2007; 8:58. 17605809 10.1186/1471-2199-8-58 PMC1931443

[65] Davey C.A. , Sargent D.F., Luger K., Maeder A.W., Richmond T.J. Solvent mediated interactions in the structure of the nucleosome core particle at 1.9 a resolution. J. Mol. Biol. 2002; 319:1097–1113. 12079350 10.1016/S0022-2836(02)00386-8

