# Supplementary material for: The conserved sex regulator DMRT1 recruits SOX9 in sexual cell fate reprogramming
Source: Nucleic Acids Res. 2021 Jun 7;49(11):6144–64. doi: 10.1093/nar/gkab448 (PMC8216462; doi:10.1093/nar/gkab448)
Supplement: gkab448_Supplemental_Files [file gkab448_supplemental_files.zip › Lindeman 2021 Supplemental Figures and Legends.pdf]

Supplemental Figure 1

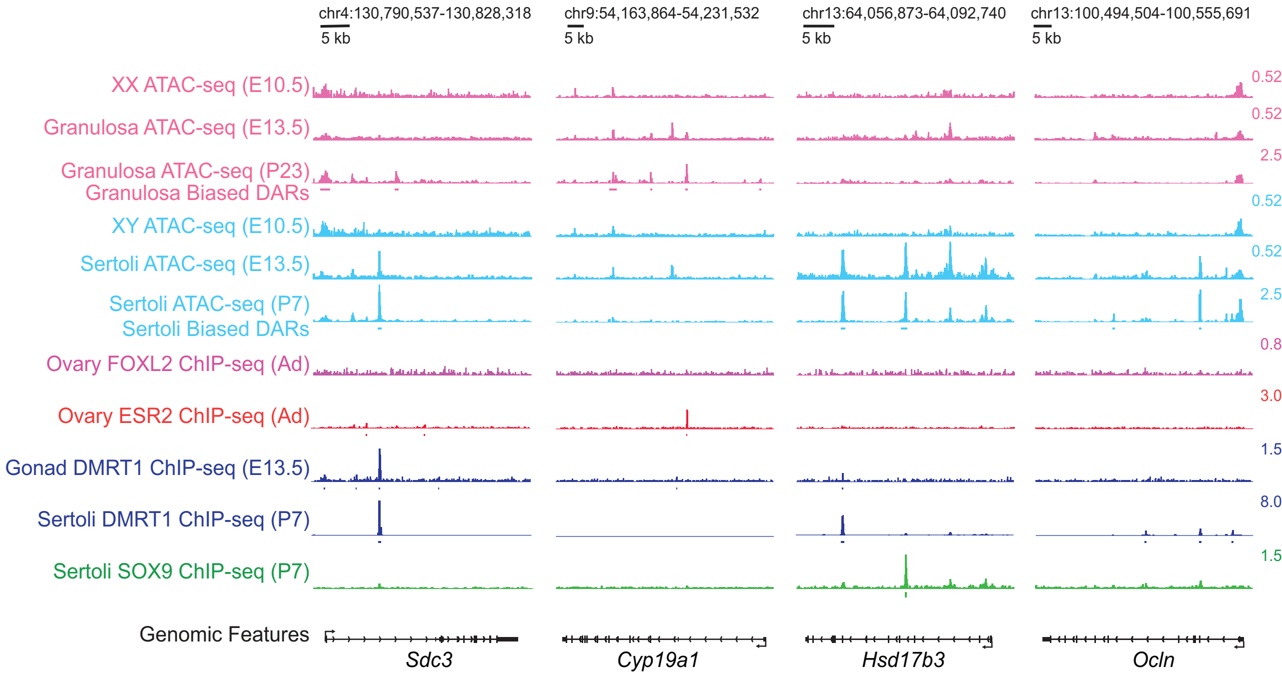

Supplemental Figure 1. Chromatin accessibility and regulatory factor binding at female- and male-biased genes. ATAC-seq and ChIP-seq data are shown for regions containing coding sequences of the female-biased genes *Sdc3* and *Cyp19a1* and the male-biased genes *Hsd17b3* and *Ocln*. *Ocln* has previously been shown to have *Dmrt1*-dependent expression (66). For ATAC-seq and ChIP-seq data, the scale shown at right indicates the number of reads per million reads sequenced for the full height of the track. Coordinates in the GRCm38 genome build are shown at the top of each panel and gene models are diagrammed at bottom.

Supplemental Figure 2

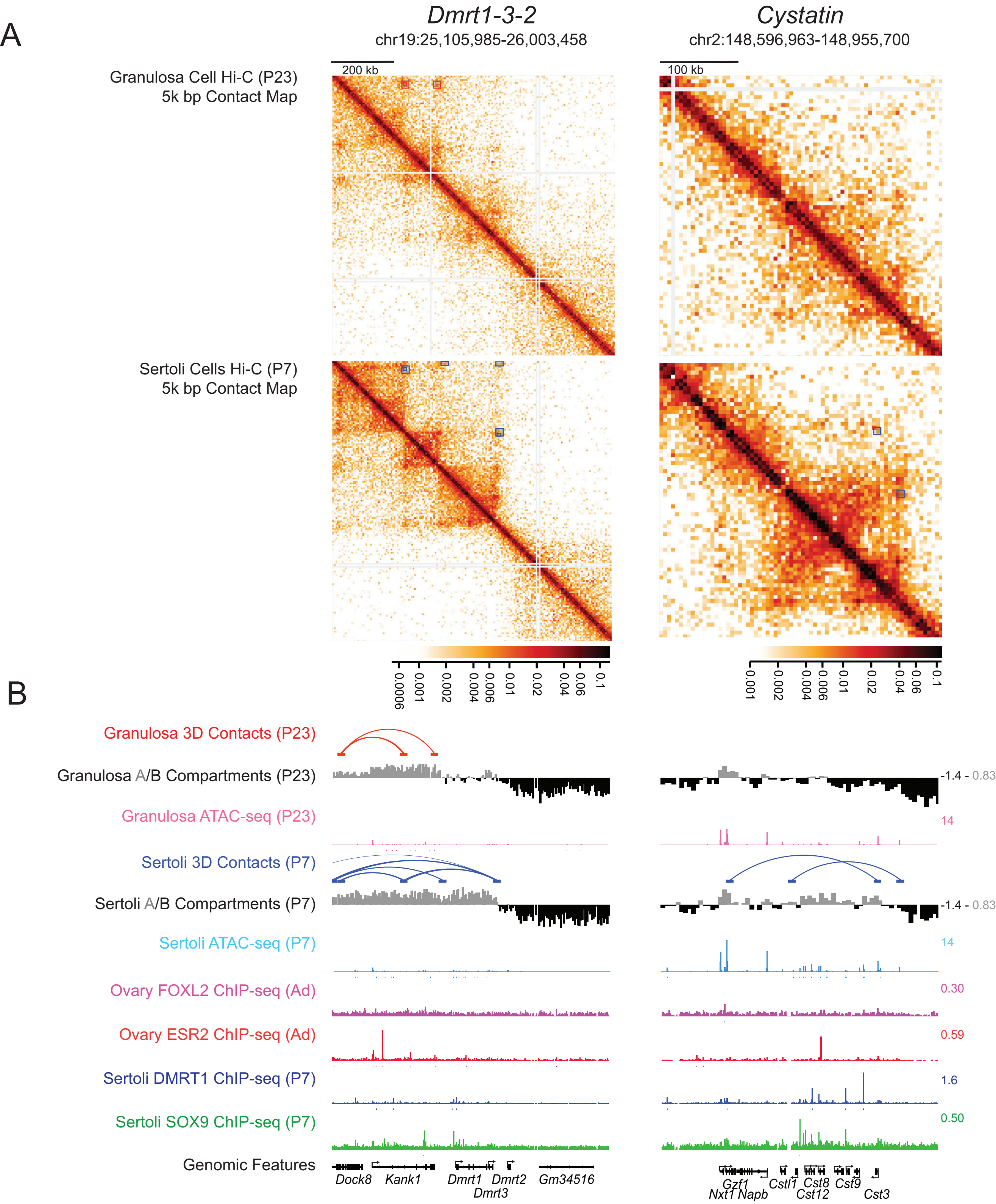

Supplemental Figure 2. Three dimensional genome organization at *Dmrt1-3-2* and *Cystatin* gene clusters in female and male somatic cells. A) Hi-C contact maps at 5kb resolution for postnatal granulosa cells (p23) and Sertoli Cells (p7). The left hand column shows a region ~900 kb surrounding the *Dmrt1-3-2* gene cluster. The right hand column shows ~350 kb surrounding the Sertol-biased *Cyst* genes. B) Accessibility and regulator binding at the same loci shown in panel A. Differentially observed loops in granulosa cells or Sertoli cells using 10 kb or 25 kb binning of the contact maps are shown as linked magenta or blue boxes respectively. A/B compartments calculated from the maps, ATAC-seq, ChIP-seq data for FOXL2, ESR2, DMRT1 and SOX9, and genomic features are shown below each contact map. A compartments are coded gray and B compartments are coded black. For ATAC-seq and ChIP-seq data, the scale shown at right indicates the number of reads per million reads sequenced for the full height of the track. Sertoli-biased A compartments and ATAC-seq accessibility are present at *Dmrt1* and the *Cyst* cluster.

Supplemental Figure 3

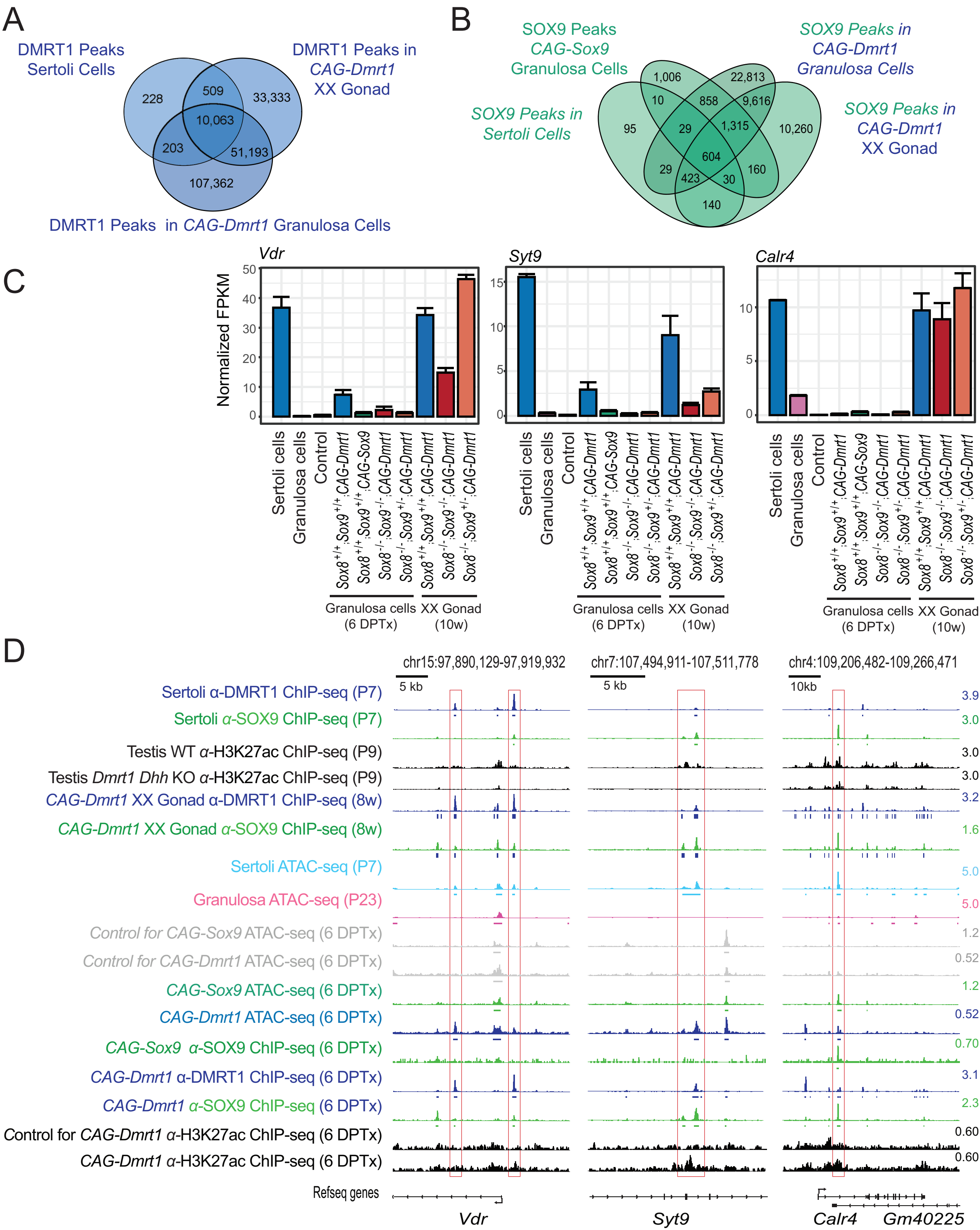

Supplemental Figure 3. Differential response to DMRT1 and SOX9 in cultured granulosa cells. A) Venn diagram comparing DMRT1 binding in Sertoli cells versus XX gonads or granulosa cells expressing ectopic DMRT1. **B)** Venn diagram comparing SOX9 binding in Sertoli cells versus XX gonads or granulosa cells expressing ectopic SOX9. **C)** Dependence of Sertoli-biased gene expression on *Sox8/9* in DMRT1-expressing XX gonads and granulosa cells. Expression levels from RNA-seq are shown for *Vdr*, *Syt9* and *Calr4*. *Vdr* can be fully activated in XX gonads if a single *Sox9* allele is present but requires both *Sox8* and *Sox9* for activation in culture, while *Syt9* activation in vivo and in culture requires both *Sox8* and *Sox9*. *Calr4* can be activated regardless of Sox8/9 dosage in XX gonads but does not respond in culture. **D)** Chromatin accessibility, histone modification and regulator binding at *Vdr*, *Syt9* and *Calr4* in wild type Sertoli and granulosa cells and cultured granulosa cells ectopically expressing DMRT1 or SOX9. Boxed regions indicate Sertoli-biased DARs. DARs in *Vdr* and *Syt9* require DMRT1 expression for SOX9 binding, while that in *Calr4* normally is bound by SOX9 and not DMRT1 in Sertoli cells and can be bound by SOX9 when it is expressed alone in granulosa cells. For ATAC-seq and ChIP-seq data, the scale shown at right indicates the number of reads per million reads sequenced for the full height of the track.
